# Higher vasoactive usage despite hemodynamic goals is associated with higher mortality in acute myocardial infarction-related cardiogenic shock

**DOI:** 10.3389/fcvm.2025.1461714

**Published:** 2025-02-13

**Authors:** Jorge A. Ortega-Hernández, Héctor González-Pacheco, Diego Araiza-Garaygordobil, Rodrigo Gopar-Nieto, Daniel Sierra-Lara-Martínez, Daniel Manzur-Sandoval, José Luis Briseño-De-La-Cruz, Salvador Mendoza-García, Álvaro Montañez-Orozco, Arturo Arzate-Ramírez, José Omar Arenas-Díaz, César A. Gómez-Rodríguez, Hector Antonio Santos-Alfaro, Jaime Hernández-Montfort, Alexandra Arias-Mendoza

**Affiliations:** ^1^ Coronary Care Unit, Instituto Nacional de Cardiología Ignacio Chávez, Tlalpan, Ciudad De México, México; ^2^ Interventional Cardiology Unit, Instituto Nacional de Cardiología Ignacio Chávez, Tlalpan, Ciudad De México, México; ^3^ Heart Failure and Recovery Program, Baylor Scott & White Health, Round Rock, TX, United States

**Keywords:** cardiogenic shock, acute myocardial infarction, vasoactive drugs, hemodynamic goals, mortality, personalized medicine

## Abstract

**Background:**

Cardiogenic shock (CS) is a severe complication of acute myocardial infarction (AMI) with high mortality. Few studies have examined the selection and subsequent choice of vasoactive agents in CS. This study investigates the impact of vasoactive drug use and in-hospital outcomes among AMI-CS.

**Materials and methods:**

A total of 309 patients who underwent pulmonary artery catheterization between 2006 and 2021 were categorized by the number of vasoactive drugs used (0–1, 2, or >2). Clinical and 24 h hemodynamic data were analyzed. Primary outcomes explored the correlation between vasoactive use and in-hospital mortality. Secondary analyses assessed hemodynamic changes and estimated mortality probabilities at different intervals using logistic regression.

**Results:**

In total, 57 patients received 0–1, 76 received 2, and 176 received >2 vasoactive drugs. The median age was 61 years; most were men (82%), and 82.8% had ST-segment elevation myocardial infarction. End-organ function showed progressive deterioration with escalating vasoactive use. Survival analysis revealed an increased mortality in the >2 vasoactive group [HR_adj_ = 4.62 (2.07–10.32)], achieving ≥5/6 hemodynamic goals that did not mitigate mortality [HR_adj_ = 7.18 (1.59–32.39)]. Subgroup analyses within patients who reached different hemodynamic goals reiterated adverse outcomes associated with >2 vasoactives (*P* < 0.05). Further analysis showed that vasopressin was associated with the highest mortality in a time-dependent fashion [HR_Day1_, 8.77 (6.04–12.75) → HR_Day30_, 1.23 (0.8–1.87)], and levosimendan had similar behavior [HR_Day1_, 2.67 (1.82–3.91) → HR_Day30_, 0.66 (0.42–1.03)].

**Conclusions:**

A significant association between the number of vasoactives and in-hospital mortality was found in AMI-CS, which requires future long-term studies to explore the role of vasoactive drug therapies and early temporary mechanical circulatory support.

## Graphical Abstract

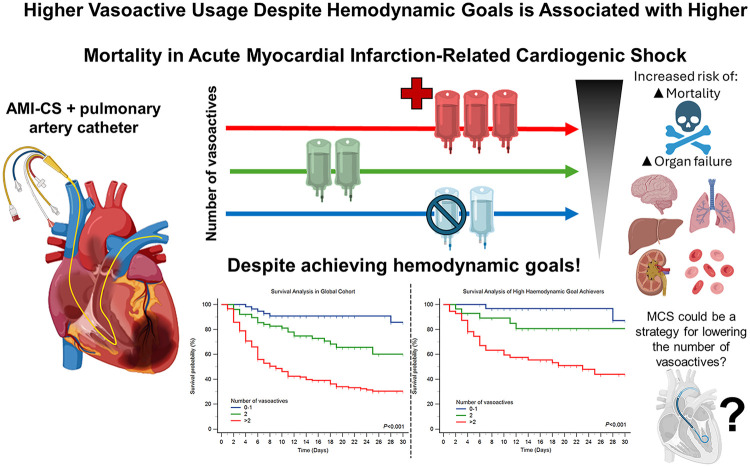

In 309 cases with acute myocardial infarction-related cardiogenic shock (AMI-CS) with pulmonary artery catheter, the higher utilization of vasoactive agents, even in the attainment of defined hemodynamic goals, emerges as a predictor of elevated mortality in the context of AMI-CS. This intriguing association is further underscored by a parallel escalation in organ dysfunction, imparting clinical significance to the delicate balance between vasoactive usage and patient outcomes.

## Background

Despite advancements in the treatment of most heart diseases, cardiogenic shock (CS) remains a severe multisystem syndrome with high morbidity and mortality rates. The primary cause of CS is coronary heart disease, which complicates 5%–12% of acute myocardial infarction (AMI) cases and leads to an in-hospital mortality rate of >40% in many cases. Several variables contribute to these high mortality rates, including pre-existing myocardial dysfunction, mechanical complications, and loss of left ventricular myocardial mass (1, 2).

Several definitions of CS exist, but most include clinical hemodynamic effects and evidence of end-organ underperfusion. Improving the identification and classification of CS phenotypes could provide a more specific guide for individualized treatments (1, 3, 4).

Recent studies using the Society for Cardiac Angiography and Interventions (SCAI) classification have shown that the use of two or more vasoactive drugs is associated with poor outcomes (5). This association was exemplified in the study by Jentzer et al., who showed that as the SCAI classification increased in severity, there was an increase in the use of vasopressors and worsening outcomes, including an increase in mortality compared with lower stages (4–7).

Few studies have examined the selection and subsequent choice of vasoactive agents in CS. However, the number of these agents and the duration of their use have been identified as risk factors and independent variables for worse outcomes in CS (4, 7). The primary objective of this study was to investigate the association between the number of vasoactive drugs used and mortality in patients with AMI-CS.

## Materials and methods

**Figure 1:**
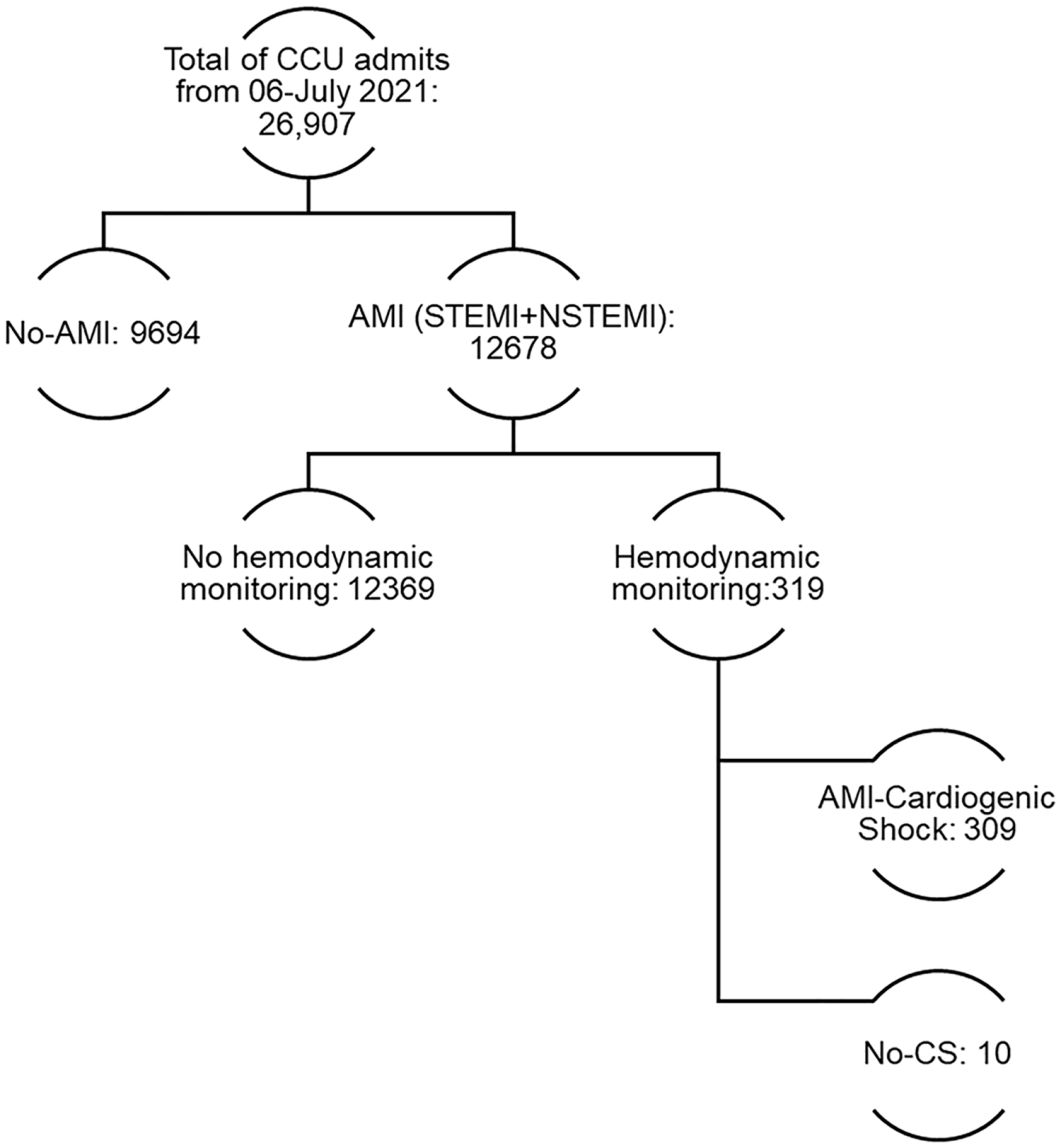
Study flowchart of CCU admissions and acute myocardial infarction cardiogenic shock invasive monitoring.

We analyzed retrospective data from 309 patients with AMI-CS and a pulmonary artery catheter (PAC) from January 2006 to July 2021 to obtain 24 h hemodynamic measurement data (Figure 1). Clinical data were obtained from electronic medical records, including patient demographics, medical history, comorbidities, laboratory results, and echocardiographic findings. The number and type of vasoactive drugs administered were recorded. The patients were categorized according to the number of vasoactive drugs used (0–1, 2, or >2). The Research and Ethics Committee approved the study protocol; patient consent was waived due to the retrospective, non-interventional nature of the study. All procedures were conducted based on the Declaration of Helsinki and local regulations.

### Classifications and group analysis

The Killip–Kimball classification was used to assess the severity of acute coronary syndrome. We used the multiorgan dysfunction syndrome (MODS) score to determine the incidence of MODS. Acute Kidney Injury (AKI) Network criteria were used to assess AKI. The 2021 SCAI (6) derived definitions were used as described previously (8, 9).

### Hemodynamic variables

Hemodynamic parameters, including cardiac index (CI), cardiac power output (CPO), cardiac power index (CPI), CPI right atrial pressure corrected (CPI_RAP_), systolic blood pressure (SBP), mean arterial pressure (MAP), pulmonary artery pulsatility index (PAPI), right atrial pressure (RAP), and pulmonary capillary wedge pressure (PCWP), were measured serially over time. PAC and derived measures were obtained using a standard formula.

### Hemodynamic high achievers

We looked at the high achievers in terms of hemodynamic measures and created an analytical high-achiever subgroup, which comprised patients who achieved 24 h values based on MAP ≥ 65 mmHg, CI ≥ 2.2 L/min/m^2^, CPI ≥ 0.32 W/m^2^, RAP < 12 mmHg, PCWP < 18 mmHg, and PAPI ≥ 1, where they achieve ≥5 of these six hemodynamic goals at 24 h. These goals were obtained based on clinical relevance and matrix analysis (for the methodology, see Supplementary Data Sheet S1).

### Missing data handling

As expected, we showed losses in some cohort measures due to delays between death and PAC installation or withdrawal, as well as any data precluded from the total 24 h measures. We used the expectation maximization algorithm to input these missing data.

### Statistical analysis

The demographic data for qualitative variables are presented in frequency and percentages, and the chi-square test or Fisher's exact test was used to assess differences. For continuous variables, median and interquartile ranges and comparison were performed using Kruskal–Wallis or Mann–Whitney *U* tests for group comparison. Bonferroni correction was used when multiple group comparisons were performed (Supplementary Tables S3, S4)

### Primary outcome

The primary analysis involved the correlation of the number of vasoactives with total in-hospital mortality. The groups and outcomes were compared against in-hospital mortality in univariate and multivariate Cox regression (age, sex, type of myocardial infarction, SCAI classification, presence of multiorgan failure, and type of reperfusion therapy). Hazard ratios and 95% confidence intervals were reported. Kaplan–Meier curves were constructed, and the log-rank test was used to assess differences.

### Secondary analysis

Patients were stratified to analyze mortality by vasoactive drug class based on the type of vasoactive drug received: dobutamine, levosimendan, norepinephrine, and vasopressin. Mortality rates were calculated for each drug class, and comparisons were made using the chi-square test for categorical variables. The groups and outcomes were compared against in-hospital mortality in univariate and multivariate Cox regression (age, sex, type of myocardial infarction, SCAI classification, presence of multiorgan failure, MCS, mechanical ventilation, and type of reperfusion therapy).

#### Hemodynamic statistical analysis

We used repeated measures analysis of variance (ANOVA) to evaluate the hemodynamic changes over time. Mauchly's test was performed for sphericity, while the Greenhouse–Geisser test was used to correct the degrees of freedom when comparing the groups. We used bivariate logistic regression to create an estimated probability of mortality against every hemodynamic measure at 0, 6, 12, and 24 h with the number of vasoactive drugs used to obtain the odds ratio (OR). A multivariate analysis was also performed to obtain the OR of these measures (Supplementary Table S5). Sensitivity analysis using the number of vasoactives as a continuous variable is presented in the Supplementary Material.

All statistical tests were two-tailed, and significance was assumed if *P* < 0.05 was obtained. Analyses were performed using IBM SPSS Statistics software (v. 22; IBM Corp., Armonk, NY, USA), MedCalc for Windows (v. 19.4; MedCalc Software, Ostend, Belgium), and SAS on Demand for Academics (SAS Institute, Cary, NC, USA).

## Results

In our cohort, 57 patients received 0–1, 76 received 2, and 176 received >2 vasoactive drugs. Most of the cohort were men (82%) with a median age of 61 (53–67) years. Among the cohort, 82.8% had ST-segment elevation myocardial infarction (STEMI) as the cause of their AMI-CS. No difference was seen in age, body mass index, diabetes, earlier heart failure, previous AMI, previous percutaneous coronary intervention (PCI), prior coronary artery bypass grafting (CABG), smoking history, out-of-hospital cardiac arrest, or type of AMI (Table 1).

**Table 1:** Demographic, clinical, and laboratory characteristics of patients stratified by vasoactive medication levels in acute myocardial infarction-related cardiogenic shock.

Variables	*N* (%)	Total, 309 (100%)	0–1 vasoactive, 57 (18.7)	2 vasoactives, 76 (24.6)	>2 vasoactives, 176 (57)	*P*-value
Gender (%)	Male	254 (82.2)	44 (77.2)	63 (82.9)	147 (83.5)	0.545
	Female	55 (17.8)	13 (22.8)	13 (17.1)	29 (16.5)	
Age (years)		61 (53–67)	59 (52–65)	62 (52–67)	62 (54–68)	0.273
BMI (kg/m^2^)		27.02 (24.34–29.39)	27.34 (25.53–30.08)	27.29 (25.03–29.3)	26.83 (24.22–29.41)	0.802
Hypertension (%)		160 (51.8)	34 (59.6)	32 (42.1)	94 (53.4)	0.108
DM2 (%)		149 (48.2)	30 (52.6)	35 (46.1)	84 (47.7)	0.739
Previous HF (%)		22 (1.8)	1 (1.8)	4 (5.3)	17 (9.7)	0.101
Previous AMI (%)		49 (15.9)	12 (21.1)	8 (10.5)	29 (16.5)	0.244
Previous PCI (%)		22 (7.1)	4 (7)	2 (2.6)	16 (9.1)	0.187
Previous CABG (%)		6 (1.9)	1 (1.8)	0 (0)	5 (2.8)	0.323
Smoking history (%)		189 (61.2)	32 (56.1)	52 (68.4)	105 (59.7)	0.293
OHCA (%)		10 (3.2)	0 (0)	3 (3.9)	7 (4)	0.328
Type of AMI (%)	NSTEMI	53 (17.2)	12 (21.1)	14 (18.4)	27 (15.3)	0.576
	STEMI	256 (82.8)	45 (78.9)	62 (81.6)	149 (84.7)	
Killip–Kimball (%)	I	46 (14.9)	11 (19.3)	15 (19.7)	20 (11.4)	<0.001
	II	89 (28.8)	23 (40.4)	19 (25)	47 (26.7)	
	III	76 (24.6)	20 (35.1)	24 (31.6)	32 (18.2)	
	IV	98 (31.7)	3 (5.3)	18 (23.7)	77 (43.8)	
Type of primary reperfusion (%)	PI	49 (15.9)	12 (21.1)	14 (18.4)	23 (13.1)	0.163
	PCI	90 (29.1)	10 (17.5)	21 (27.6)	59 (33.5)	
	NR	170 (55)	35 (61.4)	41 (53.9)	94 (53.4)	
LVEF (%)		35 (25–44)	40 (35–50)	35 (30–45)	30 (23–40)	<0.001
Hemoglobin (g/dl)		14.3 (12.6–16.2)	14.2 (12.4–15.3)	14.35 (12.7–16.55)	14.4 (12.6–16.2)	0.417
Leukocytes (cells/mm^3^)		12.9 (10.4–16.89)	11 (9.24–13.6)	14.5 (11.6–17.35)	12.9 (10.7–17.35)	0.002
Neutrophils (%)		81.4 (75–86)	78 (71.1–84.6)	80.3 (75.65–85.15)	82.1 (76.4–87)	0.095
Platelets (cells/mm^3^)		222 (176–281)	252 (199–301)	220.5 (186–292)	211 (166–272.5)	0.005
Glucose (mg/dl)		186 (130–287)	150 (120–248)	148 (115.5–243.5)	213.5 (150–299)	<0.001
BUN (mg/dl)		24.5 (17–36)	22 (16–34)	25 (16.25–32)	25 (18–39)	0.193
Creatinine (mg/dl)		1.3 (1–2)	1.1 (1–1.7)	1.2 (1–1.68)	1.5 (1.1–2.2)	0.002
eGFR (ml/min/1.73 m^2^)		55.22 (32.29–78.41)	65.43 (44.1–91.13)	61.77 (42.3–77.86)	48.51 (30.05–70.82)	0.003
Sodium (mEq/L)		136 (133–139)	136 (134–138)	136 (132–138.5)	136 (134–139)	0.729
Potassium (mEq/L)		4.3 (3.9–4.8)	4.2 (3.8–4.7)	4.3 (3.9–4.6)	4.3 (3.98–4.9)	0.366
Chloride (mEq/L)		103 (100–107)	103.7 (101–106.5)	103 (100–106)	103 (99–107)	0.876
Albumin (g/L)		3.34 (2.9–3.73)	3.46 (3.1–3.67)	3.37 (3–3.74)	3.3 (2.86–3.74)	0.691
AST (U/L)		132 (49.45–495.2)	73.75 (34.85–151)	101.75 (41–327)	259 (55.9–700)	<0.001
ALT (U/L)		77 (39.2–170)	47 (31.2–84.06)	76.6 (36.4–132)	114 (60–442)	<0.001
LDH (U/L)		747 (361–1,484)	478 (292–820)	642 (312–1,294)	1,026 (460–1,868)	<0.001
C-reactive protein (mg/L)		63 (21.4–147)	66 (24–136)	67.3 (15.75–149.5)	59.9 (21.8–150)	0.975
Maximum creatinine (mg/dl)		1.8 (1.14–2.9)	1.14 (1–2.1)	1.45 (1.057–2.5)	2.1 (1.485–3.12)	<0.001
Maximum AST (mg/dl)		215 (67–717)	85.75 (40.98–166.6)	155.5 (53–406)	471 (113–955)	<0.001
Maximum ALT (U/L)		85.7 (45–210)	50 (36–88)	77 (36.4–153)	116 (57–468.3)	<0.001
Minimum PAFI (paO2/FiO2%)		203.33 (117–300)	281.82 (247.62–342.86)	228.04 (136.47–300)	169 (100–266.67)	<0.001
Maximum 24 h lactate (mmol/L)		2.8 (1.7–5)	1.7 (1.3–2.8)	2.6 (1.6–4.1)	3.4 (2.1–5.9)	<0.001
Minimum 24 h excess base		−7.77 (−12.19−4.4)	−4.7 (−9.04, −2.14)	−6.22 (−11.86, −4.08)	−9 (−13.1, −5.98)	<0.001
Minimum 24 h pH		7.33 (7.25–7.4)	7.39 (7.35–7.44)	7.35 (7.25–7.4)	7.31 (7.23–7.37)	<0.001
Mechanical circulatory support (%)		177 (57.3)	20 (35.1)	37 (48.7)	120 (68.2)	<0.001
Angiography (%)		271 (87.7)	50 (87.7)	68 (89.5)	153 (86.9)	0.853
Number of vessels affected >50% (%)	0	12 (3.9)	3 (6)	4 (5.9)	5 (3.3)	0.176
	1	83 (26.9)	9 (18)	28 (41.2)	46 (30.1)	
	2	85 (27.5)	17 (34)	18 (26.5)	50 (32.7)	
	3	91 (29.4)	21 (42)	18 (26.5)	52 (34)	
Total PCI (%)		215 (69.6)	33 (57.9)	53 (69.7)	129 (73.3)	0.09
Dobutamine (%)		259 (83.8)	24 (42.1)	63 (82.9)	172 (97.7)	<0.001
Levosimendan (%)		98 (31.7)	1 (1.8)	11 (14.5)	86 (48.9)	<0.001
Norepinephrine (%)		248 (80.3)	10 (17.5)	64 (84.2)	174 (98.9)	<0.001
Vasopressin (%)		173 (56)	0 (0)	14 (18.4)	159 (90.3)	<0.001
Hemodialysis (%)		44 (14.2)	4 (7)	7 (9.2)	33 (18.8)	0.035
Mechanical ventilation (%)		237 (76.7)	22 (38.6)	52 (68.4)	163 (92.6)	<0.001
Total stay length (days)		12 (5–21)	17 (9–30)	15 (8–21)	8 (4–20)	<0.001
SCAI	C	41 (13.3)	19 (33.3)	14 (18.4)	8 (4.5)	<0.001
	D	152 (49.2)	27 (47.4)	36 (47.4)	89 (50.6)	
	E	116 (37.5)	11 (19.3)	26 (34.2)	79 (44.9)	
MODS score	0–4	107 (34.6)	40 (70.2)	27 (35.5)	40 (22.7)	<0.001
	5–10	111 (35.9)	15 (26.3)	33 (43.4)	63 (35.8)	
	≥11	91 (29.5)	2 (3.5)	16 (21.1)	73 (41.5)	
Multiorgan failure (%)		178 (57.6)	13 (22.8)	38 (50)	127 (72.2)	<0.001
Number of organ failures (%)	0–1	131 (42.4)	44 (77.2)	38 (50)	49 (27.8)	<0.001
	2–3	126 (40.8)	12 (21.1)	31 (40.8)	83 (47.2)	
	4–5	52 (16.8)	1 (1.8)	7 (9.2)	44 (25)	
AKI (%)		209 (67.6)	24 (42.1)	44 (57.9)	141 (80.1)	<0.001
AKIN stages (%)	0	100 (32.4)	33 (57.9)	32 (42.1)	35 (19.9)	<0.001
	I	89 (28.8)	10 (17.5)	21 (27.6)	58 (33)	
	II	43 (13.9)	6 (10.5)	8 (10.5)	29 (16.5)	
	III	77 (24.9)	8 (14)	15 (19.7)	54 (30.7)	
In-hospital mortality (%)		150 (48.5)	7 (12.3)	24 (31.6)	119 (67.6)	<0.001

AKI, acute kidney injury; ALT, alanine aminotransferase; AMI, acute myocardial infarction; AST, aspartate aminotransferase; BMI, body mass index; BUN, blood urea nitrogen; CABG, coronary artery bypass grafting; DM2, diabetes mellitus type 2; eGFR, estimated glomerular filtration rate; HF, heart failure; LDH, lactate dehydrogenase; LVEF, left ventricular ejection fraction; MODS, multiple organ dysfunction score; OHCA, out-of-hospital cardiac arrest; PAFI, ratio of arterial oxygen partial pressure to fractional inspired oxygen; PCI, percutaneous coronary intervention; PI, pharmacoinvasive strategy; SCAI, Society for Cardiovascular Angiography and Interventions; NR, non-primary reperfusion/late presenter.

The Killip–Kimball classification distribution was worse overall in the 2 and >2 vasoactive groups (*P* *<* 0.001). Specifically, the proportion of patients in Killip–Kimball class IV was higher in the >2 vasoactive group than in the other two groups (43.8% vs. 5.3% and 23.7%, respectively). Similarly, left ventricular ejection fraction (LVEF) was significantly different among the three groups, with the median LVEF decreasing as the number of vasoactive agents increased (>2, 30%; 2, 35%; 0–1, 40%; *P* *<* 0.001). In addition, higher leukocyte count, creatinine, aspartate aminotransferase, alanine aminotransferase, lactate dehydrogenase, lactate with lower platelets, estimated glomerular filtration rate, pH, and the excess base were observed as the number of drugs increased. No differences in hemoglobin, electrolytes, albumin, or C-reactive protein were found. Additionally, there were no differences in the rate or type of primary reperfusion, angiography, number of vessels, or total revascularization. The use of mechanical ventilation and hemodialysis was lower in the 0–1 vasoactive group (*P* *<* 0.001 and 0.035, respectively) (Table 1). Mechanical circulatory support was predominantly given with intra-aortic balloon pumps in one ECMO patient in the 1 vasoactive group and one Impella CP and two ECMO patients in the 3 vasoactive group.

Considering the SCAI classification, the MODS scores were higher in the >2 vasoactive group, where the presence of multiorgan failure was more common (72.2% vs. 50% and 22.8%, respectively), and the presence and degree of AKI were higher with increasing mortality in the >2 vasoactive group (67.6%), compared with the 2 (31.6%) and 0–1 (12.3%) vasoactive groups (*P* < 0.001) (Table 1).

### Serial hemodynamics according to the number of vasoactive groups

Considering the serial measures, we see that CI, CPO, CPI, and CPI_RAP_ were lower in the >2 vasoactive group compared with the 2 or 0–1 vasoactive group (*P* < 0.001). Similar behavior was seen for SBP, MAP, and PAPI, as lower values were seen in the >2 vasoactive group compared with the 2 or 0–1 vasoactive group (*P* < 0.001). In contrast, higher RAP and PCWP values were seen in the >2 vasoactive group (*P* < 0.001 and 0.009, respectively) (Tables 2, 3).

**Table 2:** Hemodynamic parameters based on vasoactive medication stratification at different time intervals.

	0–1 vasoactive	2 vasoactives	>2 vasoactives	*P*-value
0 h				
HR (bpm)	91 (82–107)	97 (85–110)	99 (84–112)	0.226
SBP (mmHg)	110 (101–124)	109 (100–116)	100 (89–116)	<0.001
MAP (mmHg)	79 (73.33–91.67)	80.5 (74.67–85.33)	74.83 (65.5–87)	0.004
RAP (mmHg)	11 (8–17)	14 (11–17)	15 (10–19)	0.038
PCWP (mmHg)	18 (12–22)	17 (14–21)	19 (15–24)	0.014
CI (L/min/m^2^)	2.76 (2.06–3.27)	2.46 (1.97–2.97)	2.09 (1.68–2.64)	<0.001
CPO (W)	0.87 (0.68–1.12)	0.8 (0.59–1.01)	0.61 (0.46–0.84)	<0.001
CPI (W/m^2^)	0.48 (0.38–0.61)	0.44 (0.33–0.53)	0.35 (0.27–0.47)	<0.001
CPI_RAP_ (W/m^2^)	0.4 (0.31–0.52)	0.37 (0.27–0.46)	0.27 (0.21–0.39)	<0.001
PAPI	1.6 (0.78–2.56)	1.04 (0.65–1.5)	0.89 (0.6–1.52)	0.004
6 h				
HR (bpm)	96 (86–106)	99.5 (89–108)	99 (89–112)	0.351
SBP (mmHg)	113 (103–120)	108.96 (95–121.5)	104.5 (95–115)	0.002
MAP (mmHg)	82.67 (75–86.67)	79.83 (72.33–86.83)	77.33 (69.33–84.01)	0.009
RAP (mmHg)	11 (7–15)	14 (10–18)	14 (10–19)	<0.001
PCWP (mmHg)	16 (12–21)	17 (12–20)	18 (14–21)	0.086
CI (L/min/m^2^)	2.93 (2.53–3.34)	2.56 (2.09–3.2)	2.32 (1.89–2.77)	<0.001
CPO (W)	0.93 (0.82–1.16)	0.8 (0.64–1.05)	0.72 (0.55–0.91)	<0.001
CPI (W/m^2^)	0.55 (0.46–0.62)	0.46 (0.35–0.6)	0.4 (0.31–0.5)	<0.001
CPI_RAP_ (W/m^2^)	0.45 (0.39–0.52)	0.38 (0.28–0.5)	0.33 (0.26–0.41)	<0.001
PAPI	1.58 (1.09–2.67)	0.97 (0.61–1.55)	1 (0.57–1.43)	<0.001
12 h				
HR (bpm)	94 (84–108)	95 (86–107)	99 (90–113)	0.128
SBP (mmHg)	112 (102–124)	108 (99–114)	102 (94–114)	<0.001
MAP (mmHg)	80.67 (76.67–90)	80 (73.33–85.33)	75.5 (68.74–84.47)	<0.001
RAP (mmHg)	11 (8–15)	13 (10–16)	14 (11–18)	0.001
PAWP (mmHg)	16 (13–21)	17 (14–20)	18 (14–21)	0.111
CI (L/min/m^2^)	2.91 (2.53–3.22)	2.71 (2.37–3.07)	2.44 (2–3.07)	<0.001
CPO (W)	0.98 (0.79–1.11)	0.85 (0.72–1.01)	0.77 (0.55–0.96)	<0.001
CPI (W/m^2^)	0.53 (0.44–0.6)	0.48 (0.4–0.56)	0.41 (0.33–0.54)	<0.001
CPI_RAP_ (W/m^2^)	0.46 (0.38–0.53)	0.4 (0.32–0.46)	0.34 (0.26–0.44)	<0.001
PAPI	1.5 (0.94–2.16)	1.17 (0.74–1.71)	1 (0.69–1.45)	<0.001
24 h				
HR (bpm)	95 (81–102)	93 (82–109)	100 (89–114)	0.005
SBP (mmHg)	110 (103–124)	107 (100–114)	102 (91–113)	<0.001
MAP (mmHg)	83.19 (73.67–88.67)	79.33 (72.5–86.33)	73.33 (67.53–83.28)	<0.001
RAP (mmHg)	12 (9–15)	13 (11–16)	14 (11–19)	<0.001
PCWP (mmHg)	16 (14–20)	16 (13–19)	18 (15–22)	0.025
CI (L/min/m^2^)	3.03 (2.5–3.46)	2.64 (2.26–3.29)	2.54 (2.04–3.15)	0.003
CPO (W)	0.99 (0.82–1.15)	0.85 (0.69–1.04)	0.79 (0.59–0.99)	<0.001
CPI (W/m^2^)	0.53 (0.43–0.65)	0.47 (0.39–0.59)	0.44 (0.34–0.54)	<0.001
CPI_RAP_ (W/m^2^)	0.46 (0.37–0.57)	0.39 (0.32–0.5)	0.35 (0.26–0.45)	<0.001
PAPI	1.61 (0.9–2.38)	1.28 (0.75–1.71)	1.07 (0.71–1.58)	0.001

CI, cardiac index; CPI, cardiac power index; CPI_RAP_, cardiac power index right atrial pressure corrected; CPO, cardiac power output; HR, heart rate; MAP, mean arterial pressure; PAPI, pulmonary artery pulsatility index; PCWP, pulmonary capillary wedge pressure; RAP, right atrial pressure; SBP, systolic blood pressure.

**Table 3:** Analysis of variance (ANOVA) for hemodynamic parameters over time and between groups.

ANOVA	Between groups (*F*, *P*)	Time (*F*, *P*)	Group*time (F*P)
HR (bpm)	2.81, 0.062	1.39, 0.248	2.03, 0.072
SBP (mmHg)	14.53, <0.001	0.43, 0.719	0.44, 0.845
MAP (mmHg)	12.63, <0.001	0.41, 0.732	0.38, 0.889
RAP (mmHg)	10.46, <0.001	1.41, 0.241	0.93, 0.47
PCWP (mmHg)	4.75, 0.009	5.71, 0.001	0.98, 0.437
CI (L/min/m^2^)	10.72, <0.001	21.35, <0.001	1.08, 0.37
CPO (W)	18.61, <0.001	13.94, <0.001	0.53, 0.768
CPI (W/m^2^)	19.96, <0.001	14.16, <0.001	0.52, 0.777
CPI_RAP_ (W/m^2^)	27.01, <0.001	13.23, <0.001	0.35, 0.898
PAPI	14.98, <0.001	0.55, 0.616	1.5, 0.187

CI, cardiac index; CPI, cardiac power index; CPI_RAP_, cardiac power index right atrial pressure corrected; CPO, cardiac power output; HR, heart rate; MAP, mean arterial pressure; PAPI, pulmonary artery pulsatility index; PCWP, pulmonary capillary wedge pressure; RAP, right atrial pressure; SBP, systolic blood pressure. *F*, *F*-values; *P*, *P*-values; *, interaction.

**Figure 2:**
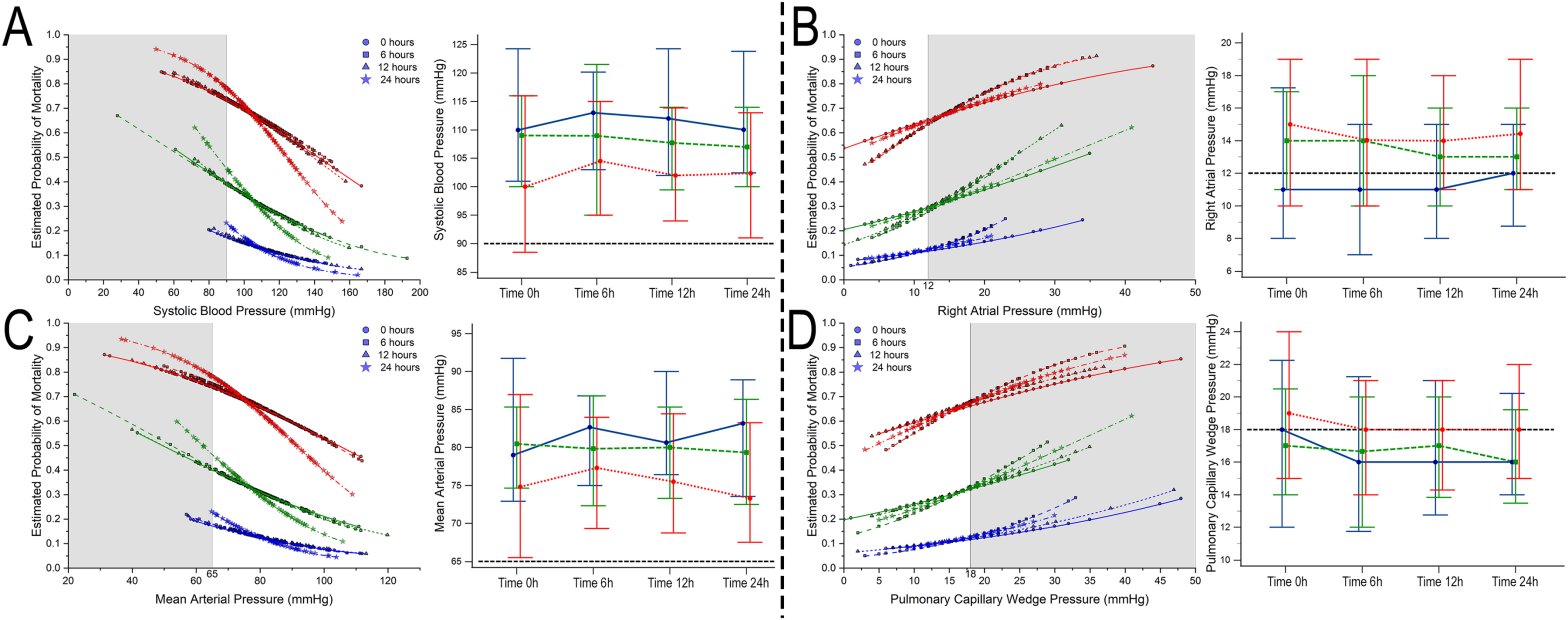
Estimated probability of mortality and time repeated measures for systolic blood pressure (**A**), right atrial pressure (**B**), mean arterial blood pressure (**C**), and pulmonary capillary wedge pressure (**D**). Blue, 0–1 vasoactive drug; green, 2 vasoactive drugs; and red, >3 vasoactive drugs.

**Figure 3:**
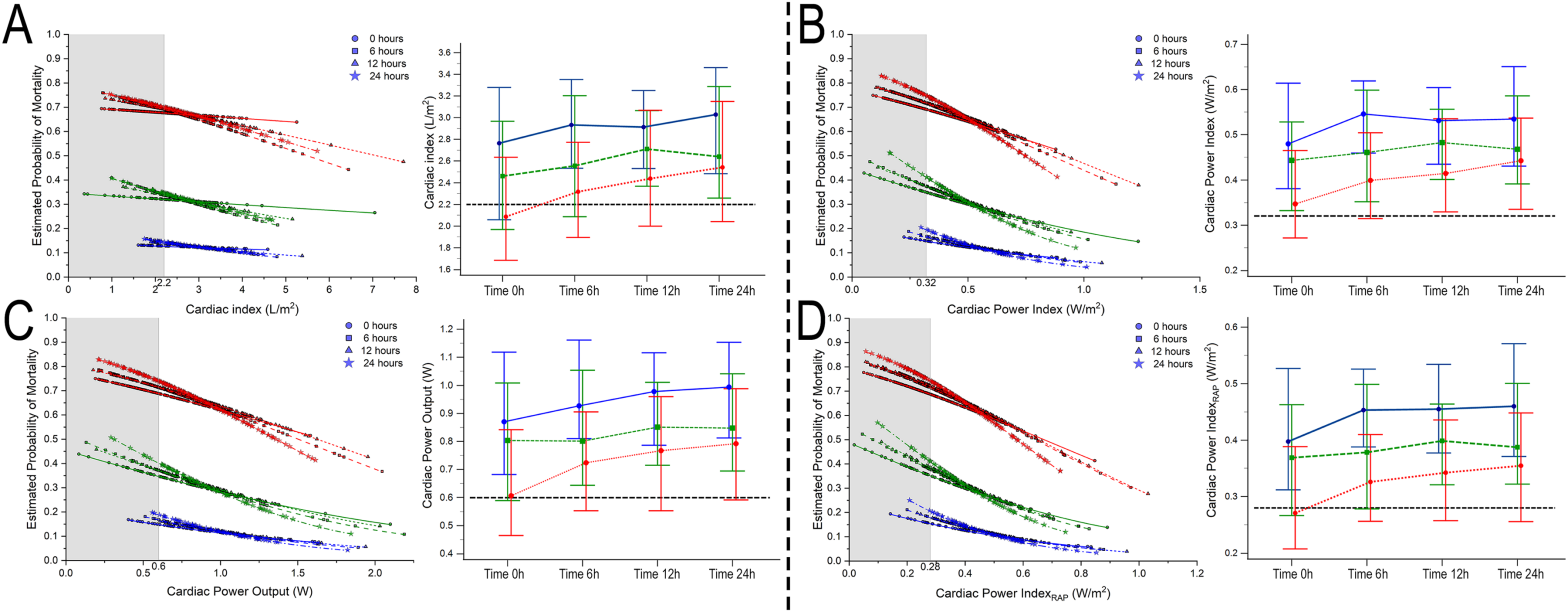
Estimated probability of mortality and time repeated measures for cardiac index (**A**), cardiac power index (**B**), cardiac power output (**C**), and cardiac power index corrected by right atrial pressure (**D**). Blue, 0–1 vasoactive drug; green, 2 vasoactive drugs; and red, >3 vasoactive drugs.

**Figure 4:**
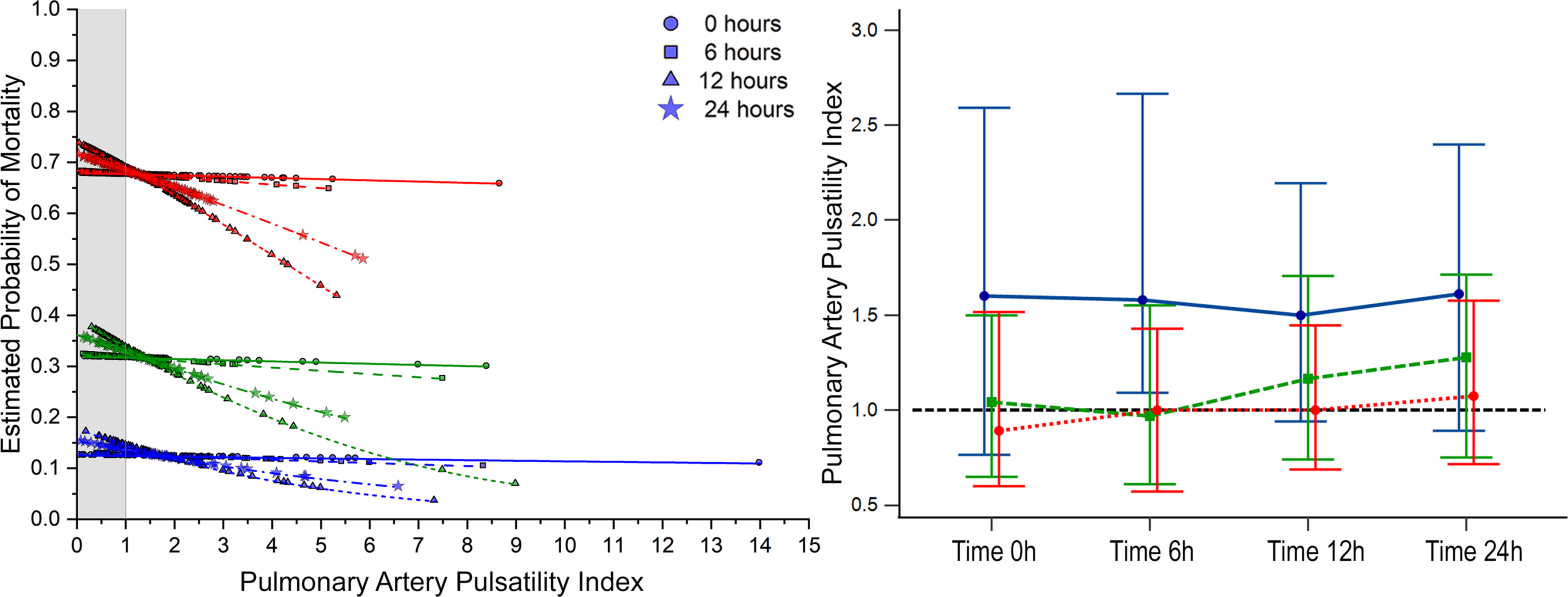
Estimated probability of mortality and time repeated measures for pulmonary artery pulsatility index. Blue, 0–1 vasoactive drug; green, 2 vasoactive drugs; and red, >3 vasoactive drugs.

Considering the univariate regression, the number of pressors was independent of predictors for mortality (*P* < 0.05 at all time points), and a higher estimated probability for mortality was seen according to the number of vasoactive drugs employed despite achieving hemodynamic goals (Figures 2–4, part not shaded in gray).

### Bivariate comparative hemodynamics in the vasoactive groups

For SBP, the ORs for ▴10 mmHg had a lower probability of death; even after adjustment, this was significant (*P* < 0.05) (Figure 2A). For MAP ▴10 mmHg, similar trends are seen as higher values are associated at all time points with lower mortality, even after the adjustment at all time points (*P* < 0.05). No significant differences were observed in CI ▴0.5 L/m^2^ between the groups at any time (*P* > 0.05) (Figure 2C). For CPO ▴0.1 W, only at 24 h did the adjusted significance reach 0.9 (0.81–0.99; *P* = 0.04) (Figure 3C). When adjusted for CPI ▴0.1 W/m^2^, only the protective OR was kept at 24 h [0.82 (0.68–0.98), *P* = 0.046] (Figure 3B). For CPI_RAP_ ▴0.1 W/m^2^, there was an association of protection at 6–24 h OR ranging from 0.81 (0.66–0.99, *P* = 0.042) to 0.82 (0.68–0.98, *P* = 0.046) (Figure 3D). For the PAPI ▴1 unit, no significance was seen at all time points (Figure 4). RAP ▴1 mmHg in the adjusted analysis at 6 and 12 h was associated with increased mortality (Figure 2B). PCWP ▴1 mmHg, when adjusted 6 and 24 h OR, has increased risk at 1.08 (1.02–1.114; *P* = 0.006) and 1.06 (1.01–1.12, *P* = 0.023), respectively (Figure 2D). In all the analyses, the number of vasoactives in the adjusted analysis shown, especially with >2 vasoactives, was an independent marker of mortality at all time points (*P* < 0.001).

Supplementary Table S5 and Table 4 provide the full unadjusted and adjusted ORs for the 0–1, 2, and >2 vasoactive groups.

**Table 4:** Adjusted bivariate relationship between hemodynamic parameters and vasoactive medications at different time intervals.

OR (95% CI; *P*-value)	0 h	6 h	12 h	24 h
Systolic blood pressure (▴10 mmHg)	0.83 (0.71–0.96; 0.016)	0.82 (0.68–0.97; 0.021)	0.79 (0.65–0.94; 0.011)	0.68 (0.56–0.82; <0.001)
0–1 vasoactive	Reference	Reference	Reference	Reference
2 vasoactives	2.29 (0.83–6.32; 0.109)	2.12 (0.77–5.88; 0.147)	2.02 (0.72–5.63; 0.18)	2 (0.71–5.66; 0.193)
>2 vasoactives	7.01 (2.64–18.59; <0.001)	6.84 (2.58–18.1; <0.001)	6.39 (2.38–17.13; <0.001)	6.11 (2.23–16.72; <0.001)
Mean arterial pressure (▴10 mmHg)	0.8 (0.64–0.98; 0.034)	0.75 (0.59–0.95; 0.02)	0.77 (0.6–0. 99; 0.041)	0.65 (0.5–0.84; 0.001)
0–1 vasoactive	Reference	Reference	Reference	Reference
2 vasoactives	2.34 (0.85–6.44; 0.099)	2.27 (0.83–6.25; 0.112)	2.14 (0.78–5.92; 0.141)	2.2 (0.79–6.14; 0.132)
>2 vasoactives	7.16 (2.71–18.91; <0.001)	7.41 (2.82–19.47: <0.001)	6.87 (2.59–18.24; <0.001)	6.79 (2.53–18.22; <0.001)
Cardiac index (▴0.5 L/m^2^)	1.02 (0.86–1.21; 0.856)	0.92 (0.77–1.09; 0.321)	0.96 (0.81–1.14; 0.636)	0.94 (0.79–1.12; 0.511)
0–1 vasoactive	Reference	Reference	Reference	Reference
2 vasoactives	2.35 (0.85–6.45; 0.099)	2.22 (0.81–6.11; 0.122)	2.3 (0.84–6.32; 0.105)	2.28 (0.83–6.25; 0.109)
>2 vasoactives	7.86 (2.96–20.9; <0.001)	7.2 (2.72–19.08; <0.001)	7.6 (2.89–19.99; <0.001)	7.49 (2.84–19.74; <0.001)
Cardiac power output (▴0.1 W)	0.97 (0.88–1.07; 0.323)	0.94 (0.85–1.02; 0.105)	0.94 (0.86–1.04; 0.225)	0.9 (0.81–0.99; 0.036)
0–1 vasoactive	Reference	Reference	Reference	Reference
2 vasoactives	2.25 (0.82–6.18; 0.116)	2.17 (0.79–5.97; 0.133)	2.23 (0.81–6.12; 0.121)	2.14 (0.78–5.9; 0.142)
>2 vasoactives	7.06 (2.65–18.85; <0.001)	6.8 (2.56–18.07; <0.001)	7.13 (2.69–18.9; <0.001)	6.69 (2.51–17.81; <0.001)
Cardiac power index (▴0.1 W/m^2^)	0.95 (0.8–1.12; 0.377)	0.89 (0.75–1.05; 0.129)	0.89 (0.75–1.06; 0.263)	0.82 (0.68–0.98; 0.046)
0–1 vasoactive	Reference	Reference	Reference	Reference
2 vasoactives	2.26 (0.82–6.2; 0.114)	2.19 (0.8–6.01; 0.129)	2.23 (0.81–6.12; 0.121)	2.14 (0.78–5.89; 0.142)
>2 vasoactives	7.15 (2.68–19.05; <0.001)	6.92 (2.61–18.34; <0.001)	7.19 (2.72–19.04; <0.001)	6.75 (2.54–17.94; <0.001)
CPI_(RAP)_ (▴0.1 W/m^2^)	0.86 (0.71–1.05; 0.152)	0.81 (0.66–0.99; 0.042)	0.83 (0.68–1.02; 0.085)	0.75 (0.6–0.94; 0.013)
0–1 vasoactive	Reference	Reference	Reference	Reference
2 vasoactives	2.22 (0.81–6.12; 0.122)	2.07 (0.75–5.72; 0.16)	2.15 (0.78–5.92; 0.138)	2.04 (0.74–5.65; 0.169)
>2 vasoactives	6.85 (2.57–18.26; <0.001)	6.41 (2.4–17.08; <0.001)	6.83 (2.57–18.13; <0.001)	6.64 (2.37–16.94; <0.001)
PAPI (▴1 unit)	1.0 (0.8–1.25; 0.996)	0.96 (0.72–1.26; 0.748)	0.79 (0.6–1.04; 0.097)	0.86 (0.63–1.17;0.342)
0–1 vasoactive	Reference	Reference	Reference	Reference
2 vasoactives	2.33 (0.85–6.41; 0.101)	2.26 (0.81–6.29; 0.12)	2.22 (0.8–6.11; 0.124)	2.29 (0.83–6.28; 0.109)
>2 vasoactives	7.75 (2.94–20.39; <0.001)	7.48 (2.79–20.06; <0.001)	7.16 (2.71–18.89; <0.001)	7.46 (2.83–19.71; <0.001)
RAP (▴1 mmHg)	2.34 (0.85–6.47; 0.101)	1.08 (1.02–1.14; 0.006)	1.06 (1.01–1.12; 0.023)	1.05 (0.99–1.11; 0.1)
0–1 vasoactive	Reference	Reference	Reference	Reference
2 vasoactives	2.34 (0.85–6.47; 0.101)	2.01 (0.72–5.59; 0.183)	2.24 (0.82–6.17; 0.118)	2.15 (0.78–5.92; 0.139)
>2 vasoactives	0.82 (2.94–20.79; <0.001)	6.56 (2.45–17.53; <0.001)	7.3 (2.76–19.27; <0.001)	7.03 (2.66–18.59; <0.001)
PCWP (▴1 mmHg)	1.02 (0.98–1.06; 0.292)	1.06 (1.02–1.12; 0.01)	1.04 (0.99–1.08; 0.116)	1.05 (1.0–1.1; 0.079)
0–1 vasoactive	Reference	Reference	Reference	Reference
2 vasoactives	2.46 (0.89–6.83; 0.084)	2.54 (0.91–7.12; 0.076)	2.52 (0.9–7.01; 0.078)	2.46 (0.89–6.78; 0.082)
>2 vasoactives	7.88 (2.98–20.87; <0.001)	8.25 (3.06–22.24; <0.001)	8.08 (3.04–21.52; <0.001)	7.98 (3.03–21; <0.001)

CPI_RAP_, cardiac power index right atrial pressure corrected; PAPI, pulmonary artery pulsatility index; PCWP, pulmonary capillary wedge pressure; RAP, right atrial pressure.

### Comparative outcomes for the vasoactive groups

**Figure 5:**
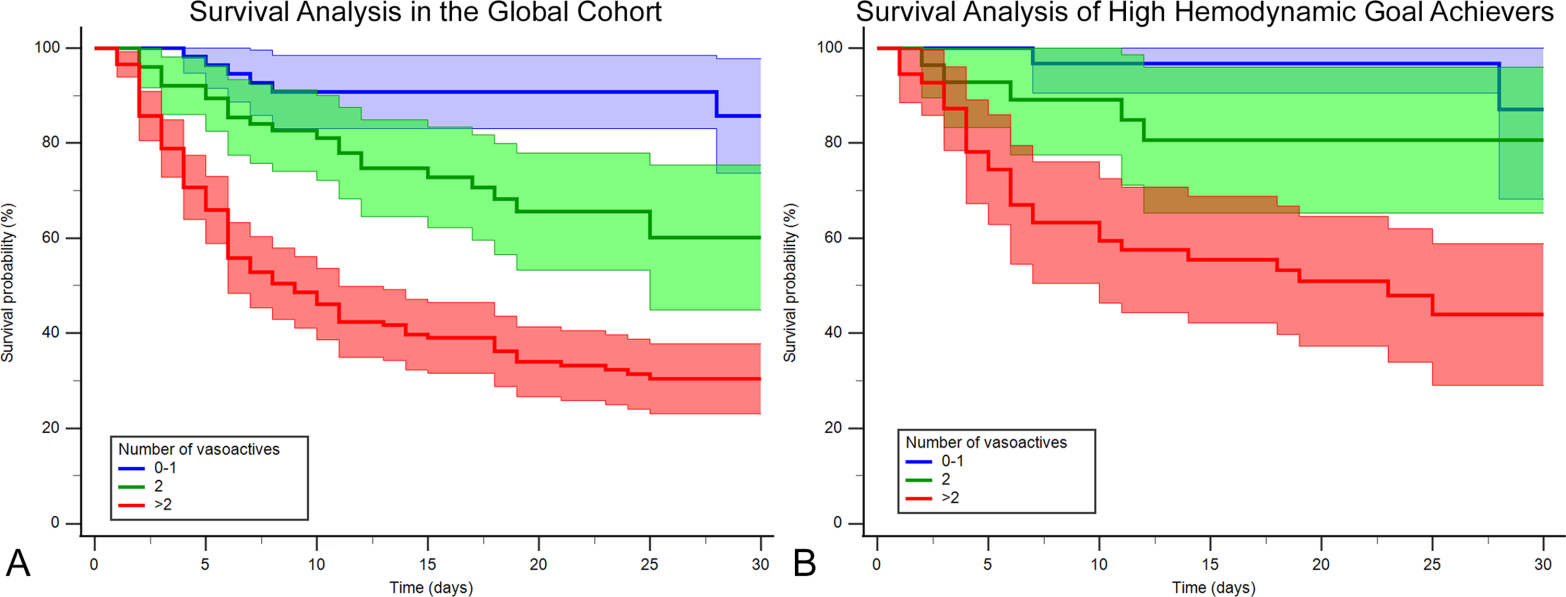
Kaplan–Meier survival curves by vasoactive groups for the (**A**) whole cohort and (**B**) hemodynamic high achievers (those who achieve ≥5/6 hemodynamic goals at 24 h: mean arterial blood pressure ≥65 mmHg, cardiac index ≥2.2 L/min/m^2^, cardiac power index ≥0.32 W/m^2^, right atrial pressure <12 mmHg, pulmonary capillary wedge pressure <18 mmHg, and pulmonary artery pulsatility index ≥1). Blue, 0–1 vasoactive drug; green, 2 vasoactive drugs; and red, >3 vasoactive drugs.

In the global cohort, we see significant differences in the Kaplan–Meier curves with a log-rank *P* < 0.001, and when compared with the 0–1 vasoactive group, patients taking two drugs had an HR of 2.83 (1.22–6.57; *P* = 0.016) and patients taking >2 drugs had an HR of 7.66 (3.57–16.4; *P* < 0.001) that is still statistically significant even after adjustment (*P* = 0.047 and <0.001, respectively) (Figure 5A).

The high-achiever subgroup (*n* = 115) also had a log-rank *P* < 0.001 (Figure 5B). When compared with the 0–1 vasoactive group, no difference was seen with patients taking two drugs, and they had an HR of 2.31 (0.55–9.7; *P* = 0.252) and an HR_adj_ of 2.12 (0.43–10.46; *P* = 0.358), but significant differences were seen when patients were taking >2 drugs, with an HR of 6.85 (3.57–22.51; *P* = 0.002) and an HR_adj_ of 7.38 (1.96–27.77; *P* = 0.003). See complete high-achiever hemodynamic analysis in Supplementary Tables S6 and S7 and Figure S2.

**Figure 6:**
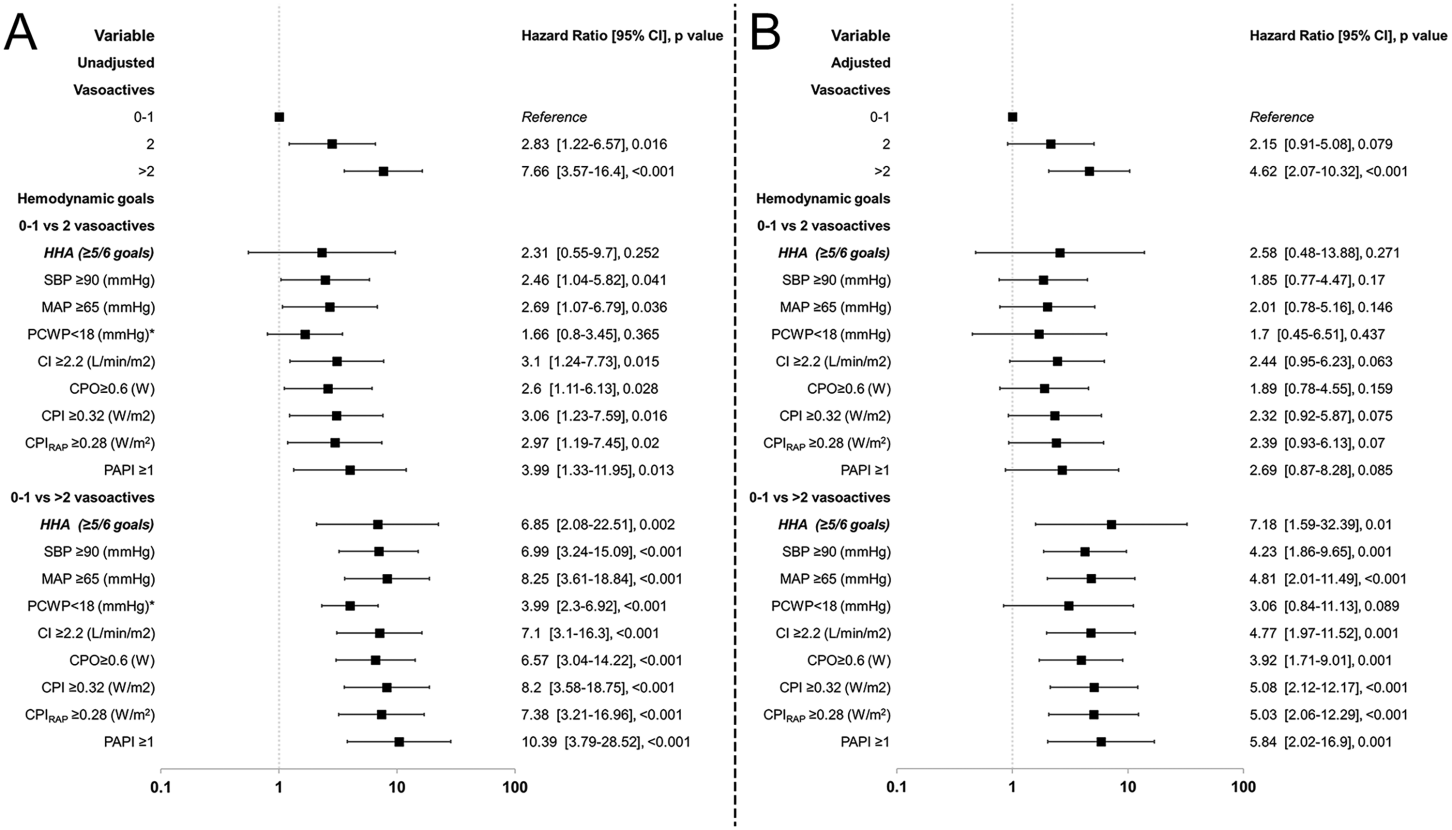
Forrest plot of hazard ratios for vasoactive groups for in-hospital mortality and hemodynamic goals compared by vasoactive groups. HHA, hemodynamic high achievers. Unadjusted (**A**) and adjusted (**B**) by selected confounders.

The Cox regression results show that when analyzing the different achiever subgroups by variable, they showed increased mortality even though they had achieved their respective goals. Although not shown in Figure 6, the RAP achievers in the 0–1 vasoactive group had a 100% survival, leading to a skewed Cox regression; the mortality rate was 14.3% in the 2 vasoactive group and 60.8% in the >2 vasoactive group. Therefore, no plot was drawn for this target subset.

Considering all the comparisons, the >2 vasoactive group showed significant differences with increased HR even after adjustment, and only in the PCWP achievers this effect was not found (*P* = 0.089). In contrast, when compared to the 2 vasoactive group, the unadjusted analysis showed an increased HR with significant *P* < 0.05, except for PCWP, but when adjusted no significance was seen (Figure 6). The PCWP also was found to have a time-dependent fashion so calculating time-specific HR at Days 1, 7, 15, and 30 demonstrates a decrease in the HR as time progresses specially in the >2 vasoactive group from 5.57 (3.1–10.02; *P* < 0.001) to 1.11 (0.59–2.09; *P* = 0.747); no significance was seen at all timepoints in the 2 vasoactive group (*P* > 0.05) (Supplementary Table S12, Figure S4).

### Comparison by drug class

Mortality by drug class was higher for every vasoactive drug used among the cohort, with a mortality rate of 53.7% with dobutamine (*P* < 0.001), 59.2% with levosimendan (*P* = 0.011), 55.2% with norepinephrine (*P* < 0.001), and 69.9% with vasopressin (*P* < 0.001).

The results of a Cox regression analysis showed that dobutamine had an HR of 1.96 (1.04–3.68; *P* = 0.038). However, no increased HR was seen for norepinephrine [HR = 1.27 (0.68–2.35); *P* = 0.448]. The proportionality was violated in levosimendan and vasopressin; for levosimendan, the HR was increased on Day 1, 2.67 (1.82–3.91, *P* < 0.001) and decreased with a time-dependent fashion on Day 30 [HR = 0.66 (0.42–1.03); *P* = 0.069]; for vasopressin, a similar trend was seen, but a higher HR was seen on Day 1 [HR = 8.77 (6.04–12.75); *P* < 0.001] with a reduction of effect in Day 30 [HR 1.23 (0.8–1.87); *P* = 0.35].

When only the high-achiever subgroup was analyzed, dobutamine [HR = 2.24 (0.45–10.31); *P* = 0.301] and norepinephrine [HR = 1.66 (0.5–5.55); *P* = 0.408] had no effect in the HR. For levosimendan and vasopressin again, a time-dependent fashion was seen with a higher HR and for more time in vasopressin [HR_Day1_ 7.61 (3.88–14.94, *P* < 0.001) to HR_Day30_ 1.3 (0.61–2.74, *P* = 0.499)] than levosimendan [HR_Day1_ 2.62 (1.22–5.65, *P* = 0.14) to a HR_Day30_ 0.86 (0.38–1.96, *P* = 0.722)]. No *P* for interaction was noticed (Supplementary Material).

Even after adjustment (not including the number of vasoactive drugs because of the risk of collinearity), vasopressin was associated with increased HR [HR = 3.39 (2.15–5.34); *P* < 0.001], while dobutamine [HR = 1.89 (1.0–3.6); *P* = 0.052], levosimendan [HR = 0.98 (0.69–1.39); *P* = 0.926], and norepinephrine [HR = 1.75 (0.96–3.17); *P* = 0.066] had a neutral effect.

This pattern was repeated in the high-achiever subgroup with vasopressin [HR = 3.0 (1.24–7.25); *P* = 0.015], dobutamine [HR = 3.13 (0.49–19.92); *P* = 0.227], and levosimendan [HR = 1.1 (0.49–2.49); *P* = 0.815]. However, it appears that in the high-achiever group, norepinephrine [HR = 3.6 (1.07–12.13); *P* = 0.039] is associated with increased risk when adjusted to confounders; no *P* for interaction was noted (Supplementary Material).

## Discussion

This study describes a direct correlation between the number of vasoactive drugs administered and in-hospital mortality, which underscores the need for a judicious and personalized approach to pharmacotherapy in patients with AMI-CS. Serial hemodynamic assessments in our study have revealed the intricacies of achieving optimal hemodynamic goals, suggesting the need to understand the physiological implications of vasoactive interventions beyond numerical hemodynamic targets. The stratification of patients based on their vasoactive drug use provides a framework for risk assessment that offers prognostic insights as in the CSWG-SCAI classification (5).

The effective management of CS demands the quick initiation of therapeutic interventions historically centered around administering vasoactive drugs. Although achieving hemodynamic goals is a clinical standard, our results show that the use of >2 vasoactive drugs significantly increases mortality risk, even among patients achieving ≥5/6 goals. This underscores that the number of vasoactive drugs may serve as a surrogate marker for disease severity and potentially nullify the benefits of achieving these targets. Meanwhile, vasoactive drugs and their prolonged and high-dose usage pose inherent risks: heightened myocardial oxygen demand, proclivity towards arrhythmias, elevated afterload, and systemic ischemia resulting from vasoconstriction, adversely impacting macro-/microcirculatory function (2, 3, 10). The use of Impella devices can cut off the dose of vasoactives; this method is proven to be effective in the DANGER trial (11) where lower mortality is seen, while VIS score can be a helpful addition, as Vallabhajosyula et al. linked that higher quartiles of VIS_peak_24 h were independently associated with higher in-hospital mortality (12).

The observed association between the administration of vasoactive drugs and in-hospital mortality underscores the pharmacotherapeutic complexity inherent in managing AMI-CS. Beyond the mere application of vasoactive agents, our study emphasizes the importance of drug selection and vigilant monitoring to achieve optimal therapeutic responses and caution when administering >2 vasoactives as this can offset the achieving hemodynamic goals. The findings prompt a critical re-evaluation of the protocols guiding vasoactive drug use and raise the question of what and how many hemodynamic goals need to be achieved and whether we want them achieved by using more vasoactive drugs weighing the increased hazard ratio. Notably, norepinephrine was associated with increased mortality in high-achiever subgroups (HR = 3.6, *P* = 0.039), suggesting that achieving hemodynamic goals with norepinephrine may come at a physiological cost that warrants careful consideration in treatment protocols.

### Hemodynamic challenges

The serial hemodynamic assessments delineating compromised cardiac function in patients receiving >2 vasoactive drugs unveil the intricacies of achieving these optimal hemodynamic goals. The juxtaposition of high achievers in hemodynamic targets with more vasoactive drugs and its relationship with adverse outcomes challenges conventional assumptions regarding the adequacy of current therapeutic strategies. However, the number of vasoactive drugs might reflect the severity of the patient's illnesses.

A previous study by Basir et al. (13) explored the role of pressures and mortality in patients with AMI-CS. However, Basir et al.'s study only explored CPO and did not perform any serial measurements. In our cohort, the repeated measures ANOVA showed the heightened risk not only with CPO and their derivatives but also all hemodynamic parameters where vasoactives were used. Despite more patients achieving the hemodynamic cut-off goal provided by the literature as an “objective,” the mortality risk was increased by the number of vasoactive drugs used. Furthermore, these patient subsets experienced worse outcomes when analyzing the >2 vasoactive group despite these patients achieving the proposed six hemodynamic goals at the 24 h time point. Moreover, we have findings similar to those of Siebert et al. (14), wherein a higher number of goals correlates with a better prognosis, but this effect is neutralized by the number of vasoactive agents used to achieve these goals in AMI-CS.

Considering the hemodynamic parameters, SBP and MAP were lower in the >2 vasoactive group. Reviews of the consensus with MAP ≥ 65 mmHg in patients with CS suggest SBP < 90 mmHg as a threshold for initiating vasoactive drug treatments (15). When subdividing by achievers in our analysis, an increased HR was seen in the 2 and >2 vasoactive groups. However, the differences statistically disappear in the 2 vasoactive group when HR is adjusted, which suggests the need to explore the appropriate number of pressors needed to maintain these goals.

For CPO and its derivates (CPI, CPI_RAP_), the same phenomenon is seen: more vasoactive drugs are associated with lower values. When considering the estimated probability of mortality, even the achievement of goals, the use of a higher “number” of vasoactive drugs is a trade-off by the risk of achieving that level.

This trend can also be observed in congestion markers, such as RAP and PCWP, with more congestion in the 2 and >2 vasoactive groups. As with PCWP, RAP gives us the phenotypes of right, left, or biventricular congested profiles, as described previously (8, 16). The novel hemodynamic goal (17) at 24 h is not achieved directly through pressure, but diuretics or mechanical ventilation plays a crucial role in this parameter. Nevertheless, optimizing cardiac output and blood pressure could help improve kidney perfusion and consequently mobilize fluids to achieve optimal urine outputs and lower congestion pressures (PCWP and RAP), thus leading to lower mortality (18). Furthermore, PCWP demonstrated a time-dependent association with mortality, indicating that early interventions to reduce congestion may mitigate these risks.

### Implications for future perspectives

Our study explored the bivariate and adjusted ORs in achieving certain hemodynamic parameter levels when SBP, MAP, or CPI_RAP_ had protective ORs at most time points, even after adjustment. These findings should be counterbalanced as the use of more pressors also increases mortality risk. The implications for risk stratification extend beyond mere mortality predictions by encompassing a holistic understanding of multiorgan dysfunction, which indicates the need for a comprehensive reassessment of therapeutic strategies. According to the proposition by Mebazaa et al. regarding refractory CS, if hemodynamic stabilization is not attained, it is advisable to contemplate using MCS with assist devices before the onset of end-organ injuries (19).

However, this study raises questions about the appropriate timing and goals for hemodynamic parameters in patients with AMI-CS. The goal of unloading pressure instead of maintaining it must be addressed for patients with CS, particularly those with AMI-CS. Various measures, such as ECMO alone, are not necessarily better, as proven by the ECLS-SHOCK study (20), although it encompasses a large population of SCAI E (by the modifier A). However, this trial proves that pressure is not the answer. The DanGer Shock trial (11, 21), among other studies, such as the STEMI Door-to-Unload (DTU) trial (22), which focused on unloading, might prove more useful as direct unloading with an Impella device will be performed. Therefore, the question may concern the appropriate timing. Recently, the DanGer Shock trial showed that upfront pre-PCI or 24 h peri-PCI use of Impella is more beneficial than conventional care in AMI-CS (11), The need for an Impella device to lower the vasopressor/inotropic drug dose had to be explored.

### Unraveling drug-specific impacts

Our study delves into the drug-specific impacts within the vasoactive pharmacopeia by elucidating the differential mortality risks associated with dobutamine, norepinephrine, and vasopressin. The heightened risk associated with vasopressin was most pronounced early in treatment (HR_Day1_ = 8.77, *P* < 0.001), with diminishing effects over time.

The oxygen-wasting effect of inotropes, such as levosimendan and dobutamine, was not observed when analyzed in animal models. While these and similar drugs correct the hemodynamic effect, their overall effect is neutral (23). Our multivariate adjustment and direct analysis findings indicate that dobutamine or levosimendan produces a neutral effect on mortality. Dobutamine was previously compared against milrinone in the DOREMI trial and with levosimendan in the SURVIVE trial, and no difference in mortality or lactate clearance was found (24–26).

In the case of vasopressin, while it might play a role in post-cardiotomy shock by mediated vasoplegia, it lowers nitric oxide production and attenuates the catecholamine resistance due to adrenergic receptor downregulation (18, 27). The unopposed peripheral vasoconstriction in patients with AMI-CS can have a deleterious profile in the pressure–volume area (conversely in the total myocardial oxygen consumption) (10), thereby putting supraphysiological stress over an already weakened cardiac output. In a porcine model, the high afterload can cause an increase in potential energy (i.e., energy-wasting) with a rightward shift of the pressure–volume loop accompanied by reduced oxygen delivery (28, 29). This was also observed by Hootman et al., where an increased OR was seen in the vasopressin group after propensity score matching (30). Traditionally, the VIS score had been linked with mortality, but in the specific cohort of AMI-CS, adjusted OR had a non-significant association [1.06 (0.73–1.55); *P* = 0.75]. In one trial (12), we hypothesize that vasopressors instead of inotropes had different implications in quantifying risk poses, and the add-on on the VIS score and specific inotrope or vasopressor score system had to be implemented in future studies. Our study also suggests that in high achievers, norepinephrine is linked to mortality; however, it is unclear whether the mechanism relates to the drug itself, or the dose used to achieve the objectives. This latter point will be explored in the NORshock (NCT05168462) trial, which will investigate the effectiveness of reduced noradrenaline in patients with CS by targeting a lower MAP of 55 mmHg compared to the standard care target MAP of ≥65 mmHg.

Beyond its immediate clinical implications, our study unveils avenues for translational research. Despite achieving their goals, the identified high-risk subgroup resistant to conventional hemodynamic optimization prompts a deeper exploration into the molecular and cellular pathways governing vasoactive drug responses.

### Strengths and limitations

This study has limitations, which primarily stem from the observational retrospective registry. This inherently limits the establishment of causation and generalizability from the results; therefore, the current conclusions presented are associations. Nevertheless, the conclusions drawn from our findings should be regarded as hypothesis-generating, and further interventional studies are required to validate and expand upon the observed associations. Another notable limitation lies in the lack of granularity regarding the exact timing of the PAC placement relative to CS diagnosis (although PAC is placed in the first 24 h of the diagnosis in our center), specific doses of vasoactives administered, coupled with the possibility of immortal time bias as the specific and intricacies of CS, are complex to disregard this effect. While the dataset captures the number of vasoactives required, the absence of information regarding individual drug dosages precludes a nuanced understanding of the potential impact of these varying doses on outcomes. In addition, this study amalgamates inotropes, such as dobutamine or levosimendan, and vasopressors, such as vasopressin and norepinephrine, in the primary analysis. These amalgamations may oversimplify the pharmacological intricacies of these agents and potentially mask nuanced effects that could influence the outcomes.

Future research endeavors should address these limitations by employing more controlled study designs and detailed pharmacokinetic assessments to reveal the precise dose–response relationships and individual contributions of distinct vasoactive agents in patients with AMI-CS.

This study boasts several strengths that enhance the robustness and significance of its findings. The employment of PAC data for serial hemodynamic assessments contributes a high level of precision in understanding the dynamic physiological changes over 24 h. This study's comprehensive approach to patient stratification based on the number of vasoactive drugs administered allows for a nuanced analysis of the impact of pharmacological interventions in patients with AMI-CS. The study incorporated advanced statistical techniques, including repeated measures ANOVA and multivariate analyses, demonstrating a sophisticated analytical framework to enhance the robustness of our conclusions. Moreover, using well-established classification systems, such as the Killip–Kimball classification and MODS scoring, adds standardization to assessing disease severity and multiorgan dysfunction. This study's consideration of missing data by applying the expectation maximization algorithm reflects a thoughtful approach to handling potential biases, further fortifying our findings' reliability. These methodological strengths position this study as a comprehensive and meticulous exploration of the intricate relationships within the complex treatment for patients with AMI-CS.

## Conclusion

A significant association between the number of vasoactive drugs and in-hospital mortality was found in AMI-CS, which requires future long-term studies to explore the role of vasoactive drug therapies and early temporary mechanical circulatory support.

## Data Availability

The raw data supporting the conclusions of this article will be made available by the authors, without undue reservation.
