# Supplementary material for: Higher vasoactive usage despite hemodynamic goals is associated with higher mortality in acute myocardial infarction-related cardiogenic shock
Source: Front Cardiovasc Med. 2025 Feb 13;12:1461714. doi: 10.3389/fcvm.2025.1461714 (PMC11865078; doi:10.3389/fcvm.2025.1461714)
Supplement: Supplementary file 1 [file Datasheet1.pdf]

Supplementary Material: Higher Vasoactive Usage Despite Hemodynamic Goals is Associated with Higher Mortality in Acute Myocardial Infarction-Related Cardiogenic Shock.

Contents

S1. Hemodynamic high-achievers 3

Table S1: Matrix correlation for hemodynamic variables 3

Table S2 & Figure S1. Cut-off points for the ROC curves to create the "Hemodynamic high-achievers" groups. 4

Table S3. Pairwise Comparison Of Demographic, Clinical, and Laboratory Characteristics of Patients Stratified by Vasoactive Medication Levels in Acute Myocardial Infarction-Related Cardiogenic Shock. 5

Table S4. Hemodynamic Parameters Based on Vasoactive Medication Stratification at Different Time Intervals Pairwise Comparison. 7

Table S5: Unadjusted Bivariate Relationship Between Hemodynamic Parameters and Vasoactive Medications at Different Time Intervals 9

Table S6: Hemodynamic differences between non-achievers and high-achievers. 11

Table S7: Analysis of Variance (ANOVA) for Hemodynamic Parameters Over Time and Between Groups Hemodynamic differences between non-achievers and high-achievers. 13

Figure S2. Survival Kaplan-Meir curves for hemodynamic high-achievers (HHA) and non-goal achievers (NGA) groups 14

Table S8. Missingness of data per variable and its percentage for Demographic, Clinical, and Laboratory Characteristics of Patients Stratified by Vasoactive Medication Levels in Acute Myocardial Infarction-Related Cardiogenic Shock. 15

Table S9. Missingness of data per variable and its percentage for the hemodynamic data in Acute Myocardial Infarction-Related Cardiogenic Shock. 16

Directed acyclic graph for the multivariable assessment (Above and below rotated for easy eye tracking Figure S3) DGA view of relevant clinical variables and it relationship with the number of vasoactives and mortality 17

Logistic regression modeling 19

Table S10. Multivariate assessment with mortality: 19

Schoenfeld Residuals Test: 20

Tables S11. Time varying analysis was carry out 1, 71 15 and 30 days for (A) PCWP, (B) Levosimendan and vasopressin and (C) HHA-levosimendan and HHA-vasopressin 21

Table S12 & Figure S4. Regression Analysis of Vasoactive Agents and Their Interaction with Time on PCWP 22

Table S13 & Figure S5. Regression Analysis of Vasoactive Agents and Their Interaction with Time on Levosimendan 23

Table S14 & Figure S6. Regression Analysis of Vasoactive Agents and Their Interaction with Time on Vasopressin 24

Table S15 & Figure S7. Regression Analysis of Vasoactive Agents and Their Interaction with Time on HHA-Levosimendan 25

Table S16 & Figure S8. Regression Analysis of Vasoactive Agents and Their Interaction with Time on HHA-Vassopresin 26

P-interaction Test: 27

Sensitivity test 28

Comparison of Sensitivity vs. Original Analysis 30

Table S18: Sensitivity test for Unadjusted Bivariate Relationship Between Hemodynamic Parameters and Vasoactive Medications at Different Time Intervals. 33

References 35

S1. Hemodynamic high achievers

For hemodynamic variables analysis, we make 6 “dynamics” theses by the clinical context in AMI-CS: Pressure, congestion, cardiac output, cardiac power, and RV power dynamics.

A matrix correlation was created, and values >0.45 were excluded, so we took non-colinear variables for each “dynamic” and chose the commonly used literature goals(1–3) for each one we selected. After the selection, these variables were coded as categories and sum for the total of 6 hemodynamic goals ROC curves and optimal cut-off point was assessed by Youden index and called high hemodynamic goal achievers’ group: Youden index J 0.244 with associated criterion ≤4 2 groups were created based on this criterion.

Table S1: Matrix correlation for hemodynamic variables

		Pressure Dynamics			Congestion Dynamics		Cardiac output Dynamics		Cardiac power Dynamics			RV power Dynamic
		**SBP**	**DBP**	**MAP**	**RAP**	**PCWP**	**CO**	**CI**	**CPO**	**CPI**	**CPI_{RAP}**	**PAPI**
**Pressure Dynamics**	**SBP**		0.50	0.83	-0.02	0.08	0.11	0.15	0.43	0.48	0.52	0.10
	**DBP**	0.50		0.90	0.02	0.07	0.11	0.07	0.45	0.44	0.49	0.08
	**MAP**	0.83	0.90		0.01	0.08	0.12	0.12	0.51	0.53	0.58	0.10
**Congestion Dynamics**	**RAP**	-0.02	0.02	0.01		0.40	-0.04	-0.06	-0.03	-0.05	-0.26	-0.51
	**PCWP**	0.08	0.07	0.08	0.40		-0.01	-0.04	0.03	0.00	-0.08	-0.09
**Cardiac output Dynamics**	**CO**	0.11	0.11	0.12	-0.04	-0.01		0.95	0.91	0.85	0.79	0.07
	**CI**	0.15	0.07	0.12	-0.06	-0.04	0.95		0.86	0.90	0.84	0.07
**Cardiac power Dynamics**	**CPO**	0.43	0.45	0.51	-0.03	0.03	0.91	0.86		0.96	0.92	0.10
	**CPI**	0.48	0.44	0.53	-0.05	0.00	0.85	0.90	0.96		0.97	0.10
	**CPI_{RAP}**	0.52	0.49	0.58	-0.26	-0.08	0.79	0.84	0.92	0.97		0.21
**RV power Dynamic**	**PAPI**	0.10	0.08	0.10	-0.51	-0.09	0.07	0.07	0.10	0.10	0.21	

CI: Cardiac Index, CPI: Cardiac Power Index, CPI_{RAP}: Cardiac Power Index Right Atrial Pressure corrected, CPO: Cardiac Power Output, MAP: Mean Arterial Pressure, PAPi: Pulmonary Artery Pulsatility Index, PCWP: Pulmonary Capillary Wedge Pressure, RAP: Right Atrial Pressure, SBP: Systolic Blood Pressure

Table S2 & Figure S1. Cut-off points for the ROC curves to create the "Hemodynamic high-achievers" groups.

Criterion	Sensitivity	Specificity	+LR	95% CI	-LR	95% CI
**<0**	0.00	100.00			1.00	1.0 - 1.0
**≤0**	4.00	100.00			0.96	0.9 - 1.0
**≤1**	10.00	98.11	5.30	1.6 - 17.9	0.92	0.9 - 1.0
**≤2**	22.67	92.45	3.00	1.6 - 5.6	0.84	0.8 - 0.9
**≤3**	42.67	76.10	1.79	1.3 - 2.5	0.75	0.6 - 0.9
**≤4**	75.33	49.06	1.48	1.2 - 1.8	0.50	0.4 - 0.7
**≤5**	94.67	22.01	1.21	1.1 - 1.3	0.24	0.1 - 0.5
**≤6**	100.00	0.00	1.00	1.0 - 1.0		

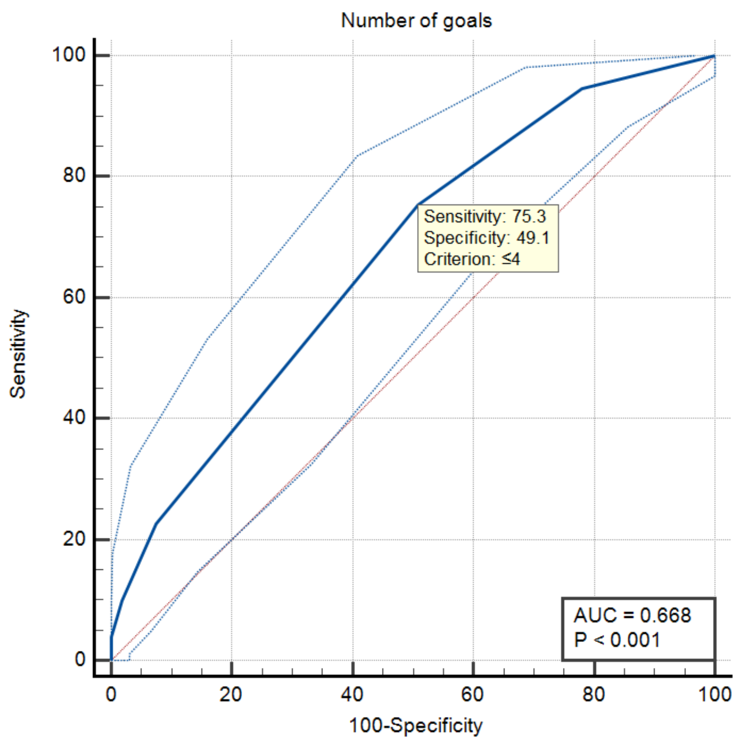

Table S3. Pairwise Comparison Of Demographic, Clinical, and Laboratory Characteristics of Patients Stratified by Vasoactive Medication Levels in Acute Myocardial Infarction-Related Cardiogenic Shock.

Variables	N (%)	1 vs. 2	1 vs.>2	2 vs.>2
Killip-Kimball (%)	I			
	II			
	III		0.023	
	IV	0.012	<0.001	0.008
Type of primary reperfusion (%)	PCI			
	PI			
	NR			
LVEF (%)		0.023	<0.001	0.113
Leucocytes (cells/mm^3)		0.003	0.006	1
Platelets (cells/mm^3)		0.269	0.004	0.476
Glucose (mg/dL)		1	0.003	0.001
Creatinine (mg/dL)		1	0.008	0.038
eGFR (ml/min/1.73m^2)		1	0.006	0.067
AST (U/L)		0.279	<0.001	0.013
ALT (U/L)		0.097	<0.001	0.001
LDH (U/L)		0.611	<0.001	0.006
Maximum creatinine (mg/dL)		0.335	<0.001	0.003
Maximum AST (mg/dL)		0.054	<0.001	<0.001
Maximum ALT (U/L)		0.12	<0.001	0.004
Minimum PAFI (paO2/FiO2%)		0.01	<0.001	0.034
Maximum 24h Lactate (mmol/L)		0.01	<0.001	0.06
Minimum 24h Excess base		0.289	<0.001	0.005
Minimum 24h pH		0.002	<0.001	0.141
Mechanical circulatory support (%)			<0.001	0.01
Dobutamine (%)		<0.001	<0.001	<0.001
Levosimendan (%)		0.034	<0.001	<0.001
Norepinephrine (%)		<0.001	<0.001	<0.001
Vasopressin (%)		<0.001	<0.001	<0.001
Hemodialysis (%)		NS	NS	NS
Mechanical ventilation (%)		0.002	<0.001	<0.001
Total stay length (days)		0.558	<0.001	0.003
SCAI	C		<0.001	0.001
	D			
	E		0.002	
MODS score	0-4	<0.001	<0.001	
	5-10			
	≥11	0.01	<0.001	0.006

Multiorgan failure (%)		0.004	<0.001	0.002
Number of organ failures (%)	0-1	0.004	<0.001	
	2-3	0.048	0.001	
	4-5		<0.001	0.013
AKI (%)			<0.001	0.001
AKIN stages (%)	0		<0.001	0.001
	I			
	II			
	III		0.04	
In hospital mortality (%)		0.028	<0.001	<0.001

AKI: Acute Kidney Injury, ALT: Alanine Aminotransferase, AST: Aspartate Aminotransferase, LVEF: Left Ventricular Ejection Fraction, LDH: Lactate Dehydrogenase, MODS: Multiple Organ Dysfunction Score, PCI: Percutaneous Coronary Intervention, PI: Pharmacoinvasive strategy, NS: Not Significant, NR: non-primary reperfused, SCAI: Society for Cardiovascular Angiography & Interventions.

Table S4. Hemodynamic Parameters Based on Vasoactive Medication Stratification at Different Time Intervals Pairwise Comparison.

	1 vs. 2	1 vs.>2	2 vs.>2
0 hours			
HR (bpm)			
SBP (mmHg)	0.689	0.001	0.038
MAP (mmHg)	1	0.016	0.042
RAP (mmHg)	0.324	0.031	1
PCWP (mmHg)	1	0.228	0.02
CI (L/min/m2)	0.228	<0.001	0.014
CPO (W)	0.15	<0.001	0.003
CPI (W/m2)	0.203	<0.001	0.002
CPI_{RAP} (W/m2)	0.156	<0.001	0.001
PAPi	0.07	0.003	1
6 hours			
HR (bpm)			
SBP (mmHg)	0.25	0.002	0.335
MAP (mmHg)	1	0.013	0.162
RAP (mmHg)	0.013	<0.001	1
PCWP (mmHg)			
CI (L/min/m2)	0.018	<0.001	0.061
CPO (W)	0.017	<0.001	0.038
CPI (W/m2)	0.026	<0.001	0.028
CPI_{RAP} (W/m2)	0.012	<0.001	0.01
PAPi	<0.001	<0.001	1
12 hours			
HR (bpm)			
SBP (mmHg)	0.089	<0.001	0.258
MAP (mmHg)	0.272	<0.001	0.102
RAP (mmHg)	0.133	0.001	0.328
PAWP (mmHg)			
CI (L/min/m2)	0.413	<0.001	0.067
CPO (W)	0.162	<0.001	0.02
CPI (W/m2)	0.137	<0.001	0.023
CPI_{RAP} (W/m2)	0.053	<0.001	0.009
PAPi	0.038	<0.001	0.648
24 hours			
HR (bpm)	1	0.017	0.044
SBP (mmHg)	0.202	<0.001	0.106
MAP (mmHg)	0.37	<0.001	0.029
RAP (mmHg)	0.03	<0.001	0.792

PCWP (mmHg)	1	0.048	0.18
CI (L/min/m2)	0.187	0.002	0.51
CPO (W)	0.075	<0.001	0.108
CPI (W/m2)	0.096	<0.001	0.096
CPI_{RAP} (W/m2)	0.025	<0.001	0.049
PAPi	0.044	0.001	0.985

CI: Cardiac Index, CPI: Cardiac Power Index, CPI_{RAP}: Cardiac Power Index Right Atrial Pressure corrected, CPO: Cardiac Power Output, HR: Heart Rate, MAP: Mean Arterial Pressure, PAPi: Pulmonary Artery Pulsatility Index, PCWP: Pulmonary Capillary Wedge Pressure, RAP: Right Atrial Pressure, SBP: Systolic Blood Pressure

Table S5: Unadjusted Bivariate Relationship Between Hemodynamic Parameters and Vasoactive Medications at Different Time Intervals.

OR (95 %CI; *P*-value)	0 hours	6 hours	12 hours	24 hours
Systolic Blood Pressure (▲10 mmHg)	0.82 (0.72-0.95; 0.007)	0.83 (0.71-0.97; 0.019)	0.81 (0.68-0.96; 0.014)	0.69 (0.58-0.82; < 0.001)
0-1 vasoactives	Reference	Reference	Reference	Reference
2 vasoactives	3.08 (1.21-7.82; 0.018)	3.04 (1.19-7.7; 0.02)	2.92 (1.14-7.45; 0.025)	2.78 (1.08-7.16; 0.034)
> 2 vasoactives	13.01 (5.51-30.71; < 0.001)	13.43 (5.7-31.64; < 0.001)	12.64 (5.34-29.91; < 0.001)	11.95 (5.02-28.45; < 0.001)
Mean Arterial Pressure (▲10 mmHg)	0.77 (0.63-0.93; 0.009)	0.75 (0.6-0.92; 0.007)	0.77 (0.61-0.96; 0.022)	0.62 (0.48-0.78; < 0.001)
0-1 vasoactives	Reference	Reference	Reference	Reference
2 vasoactives	3.21 (1.6-8.71; 0.014)	3.15 (1.24-8.04; 0.016)	3.04 (1.19-7.73; 0.02)	2.94 (1.15-7.54; 0.025)
> 2 vasoactives	13.49 (5.72-31.82; < 0.001)	13.8 (5.86-32.5; < 0.001)	13.05 (5.53-30.82; < 0.001)	12.18 (5.13-28.92; < 0.001)
Cardiac index (▲0.5 L/m^2)	0.97 (0.83-1.14; 0.73)	0.89 (0.76-1.03; 0.114)	0.92 (0.79-1.07; 0.285)	0.9 (0.77-1.05; 0.171)
0-1 vasoactives	Reference	Reference	Reference	Reference
2 vasoactives	3.25 (1.8-8.24; 0.013)	3.05 (1.2-7.76; 0.019)	3.2 (1.27-8.11; 0.014)	3.16 (1.25-8.02; 0.015)
> 2 vasoactives	14.47 (6.08-34.46; < 0.001)	13.27 (5.6-31.44; < 0.001)	14.162 (6.02-33.34; < 0.001)	13.97 (5.94-32.9; < 0.001)
Cardiac power output (▲0.1 W)	0.93 (0.85-1.01; 0.092)	0.9 (0.83-0.98; 0.017)	0.92 (0.84-1; 0.046)	0.87 (0.8-0.95; 0.003)
0-1 vasoactives	Reference	Reference	Reference	Reference
2 vasoactives	3.07 (1.21-7.8; 0.019)	2.94 (1.15-7.5; 0.024)	3.05 (1.2-7.74; 0.019)	2.9 (1.14-7.41; 0.026)
> 2 vasoactives	12.7 (5.35-30.34; < 0.001)	12.34 (5.2-29.3; < 0.001)	12.93 (5.46-30.61; < 0.001)	12.26 (5.18-29.04; < 0.001)
Cardiac power index (▲0.1 W/m^2)	0.88 (0.75-1.04; 0.128)	0.84 (0.72-0.98; 0.03)	0.86 (0.72-1.01; 0.062)	0.78 (0.65-0.92; 0.003)
0-1 vasoactives	Reference	Reference	Reference	Reference
2 vasoactives	3.09 (1.22-7.85; 0.018)	2.97 (1.16-7.57; 0.023)	3.05 (1.2-7.76; 0.019)	2.91 (1.14-7.42; 0.026)
> 2 vasoactives	12.91 (5.42-30.77; < 0.001)	12.5 (5.26-29.68; < 0.001)	13.04 (5.51- 30.86; < 0.001)	12.27 (5.18-29.06; < 0.001)
$CPI_{(RAP)}$ (▲0.1 W/m^2)	0.82 (0.68-0.98; 0.031)	0.78 (0.65-0.92; 0.005)	0.76 (0.64-0.93; 0.008)	0.7 (0.57-0.85; < 0.001)
0-1 vasoactives	Reference	Reference	Reference	Reference
2 vasoactives	2.99 (1.17-7.61; 0.022)	2.78 (1.09-7.13; 0.033)	2.88 (1.13-7.35; 0.027)	2.7 (1.05-6.93; 0.039)
> 2 vasoactives	12.1 (5.08-28.86; < 0.001)	11.49 (4.82-27.41; < 0.001)	11.9 (5-28.3; < 0.001)	11.18 (4.69-26.63; < 0.001)

PAPI (▲ 1 unit)	0.99 (0.81-1.19; 0.912)	0.97 (0.743-1.245; 0.813)	0.79 (0.6-1.01; 0.068)	0.86 (0.5-1.12; 0.277)
0-1 vasoactives	Reference	Reference	Reference	Reference
2 vasoactives	3.27 (1.29-8.34; 0.013)	3.21 (1.24-8.32; 0.016)	3.01 (1.18-7.67; 0.021)	3.11 (1.22-7.92; 0.017)
> 2 vasoactives	14.78 (6.23-35.1; < 0.001)	14.47 (5.97-35.07; < 0.001)	13.16 (5.57-31.1; < 0.001)	13.87 (5.87-32.76; < 0.001)
RAP (▲1 mmHg)	1.04 (1-1.08; 0.048)	1.08 (1.03-1.14; 0.002)	1.08 (1.03-1.13; 0.003)	1.05 (1-1.1; 0.058)
0-1 vasoactives	Reference	Reference	Reference	Reference
2 vasoactives	3.17 (1.25-8.05; 0.015)	2.73 (1.07-7.01; 0.037)	2.94 (1.15-7.5; 0.024)	2.92 (1.14-7.46; 0.025)
> 2 vasoactives	14.22 (6.04-33.46; < 0.001)	12.36 (5.22-29.27; < 0.001)	12.5 (5.28-29.6; < 0.001)	13.1 (5.54-30.98; < 0.001)
PCWP(▲1 mmHg)	1.04 (1-1.08; 0.047)	1.07 (1.02-1.12; 0.003)	1.04 (1-1.09; 0.054)	1.05 (1.01-1.1; 0.022)
0-1 vasoactives	Reference	Reference	Reference	Reference
2 vasoactives	3.59 (1.4-9.18; 0.008)	3.46 (1.35-8.88; 0.01)	3.45 (1.35-8.8; 0.01)	3.36 (1.32-8.53; 0.011)
> 2 vasoactives	14.77 (6.26-34.85; < 0.001)	14.78 (6.22-35.12; < 0.001)	14.97 (6.34-35.35; < 0.001)	14.36 (6.1-33.78; < 0.001)

CPI_{RAP}: Cardiac Power Index Right Atrial Pressure corrected, PAPI: Pulmonary Artery Pulsatility Index, PCWP: Pulmonary Capillary Wedge Pressure, RAP: Right Atrial Pressure

Table S6: Hemodynamic differences between non-achievers and high-achievers.

	**Non-Achievers (n=194)**	**High-Achievers (n=115)**	**P-value**
0 hours			
HR (bpm)	98 (84-112)	97 (84-110)	0.673
SBP (mmHg)	103 (92-116)	109 (98-120)	0.023
MAP (mmHg)	76.67 (67.33-88)	79.33 (70-86.67)	0.302
RAP (mmHg)	16 (12-19)	10 (8-14)	< 0.001
PCWP (mmHg)	20 (16-24)	16 (12-20)	< 0.001
CI (L/min/m^2)	2.19 (1.79-2.78)	2.49 (1.87-3.21)	0.026
CPO (W)	0.68 (0.48-0.89)	0.73 (0.55-1)	0.082
CPI (W/m^2)	0.38 (0.29-0.49)	0.42 (0.3-0.53)	0.059
CPI_{RAP} (W/m^2)	0.3 (0.21-0.4)	0.37 (0.26-0.47)	0.001
PAPI	0.83 (0.5-1.38)	1.36 (0.9-2.56)	< 0.001
6 hours			
HR (bpm)	99 (87-111)	98 (89-110)	0.672
SBP (mmHg)	105 (95-119)	109 (99-118)	0.227
MAP (mmHg)	78 (70-85)	79 (71.29-84.67)	0.747
RAP (mmHg)	16 (12-19)	10 (8-14)	< 0.001
PCWP (mmHg)	18 (15-22)	14 (11-19)	< 0.001
CI (L/min/m^2)	2.4 (1.92-2.98)	2.63 (2.21-3.15)	0.006
CPO (W)	0.77 (0.58-0.99)	0.82 (0.68-1.04)	0.052
CPI (W/m^2)	0.42 (0.32-0.56)	0.46 (0.38-0.55)	0.041
CPI_{RAP} (W/m^2)	0.34 (0.26-0.45)	0.4 (0.32-0.48)	0.002
PAPI	0.91 (0.5-1.31)	1.5 (0.94-2.4)	< 0.001
12 hours			
HR (bpm)	99 (87-113)	95 (86-103)	0.048
SBP (mmHg)	104 (96-114)	108 (98-116)	0.252
MAP (mmHg)	78.17 (70.33-85.33)	78.95 (72-86)	0.476
RAP (mmHg)	15 (12-19)	10 (8-13)	< 0.001
PAWP (mmHg)	18 (15-22)	15 (11-19)	< 0.001
CI (L/min/m^2)	2.6 (2.06-3.06)	2.78 (2.36-3.22)	0.03
CPO (W)	0.81 (0.62-0.99)	0.85 (0.71-1.03)	0.059
CPI (W/m^2)	0.44 (0.35-0.55)	0.49 (0.4-0.58)	0.031
CPI_{RAP} (W/m^2)	0.36 (0.27-0.45)	0.41 (0.33-0.51)	< 0.001
PAPI	0.95 (0.63-1.34)	1.5 (1-2.33)	< 0.001
24 hours			
HR (bpm)	99 (87-112)	95 (83-107)	0.045
SBP (mmHg)	103 (92-114)	107 (100-118)	0.022
MAP (mmHg)	75.67 (68.33-84.33)	79.33 (71.67-86.33)	0.032
RAP (mmHg)	16 (13-19)	10 (8-12)	< 0.001
PCWP (mmHg)	19 (17-23)	14 (12-16)	< 0.001
CI (L/min/m^2)	2.5 (2.05-3.06)	2.91 (2.44-3.35)	< 0.001

CPO (W)	0.79 (0.59-1.01)	0.9 (0.75-1.15)	<0.001
CPI (W/m^2)	0.43 (0.33-0.55)	0.51 (0.43-0.64)	< 0.001
CPI_{RAP} (W/m^2)	0.34 (0.25-0.45)	0.43 (0.37-0.54)	< 0.001
PAPI	0.92 (0.6-1.33)	1.61 (1.26-2.41)	< 0.001
Vasoactive groups			
0-1	25 (12.9)	32 (27.8)	0.003
2	48 (24.7)	28 (24.3)	
>2	121 (62.4)	55 (47.8)	
In-hospital mortality (%)	113 (58.2)	37 (32.2)	< 0.001

AKI: Acute Kidney Injury, ALT: Alanine Aminotransferase, AMI: Acute Myocardial Infarction, AST: Aspartate Aminotransferase, BMI: Body Mass Index, BUN: Blood Urea Nitrogen, CABG: Coronary Artery Bypass Grafting, DM2: Diabetes Mellitus type 2, eGFR: estimated Glomerular Filtration Rate, HF: Heart Failure, LDH: Lactate Dehydrogenase, LVEF: Left Ventricular Ejection Fraction, MODS: Multiple Organ Dysfunction Score, OHCA: Out-of-Hospital Cardiac Arrest, PAFI: ratio of arterial oxygen partial pressure to fractional inspired oxygen, PCI: Percutaneous Coronary Intervention, PI: Pharmacoinvasive strategy, SCAI: Society for Cardiovascular Angiography and Interventions

Table S7: Analysis of Variance (ANOVA) for Hemodynamic Parameters Over Time and Between Groups Hemodynamic differences between non-achievers and high-achievers.

**ANOVA**	**Between groups (*F, P*)**	**Time (*F, P*)**	**Group*Time (*F*P*)**
HR (bpm)	2, 0.158	1.24, 0.294	2.47, 0.072
SBP (mmHg)	4.97, 0.027	0.75, 0.518	0.55, 0.637
MAP (mmHg)	1.69, 0.195	0.063, 0.588	1.18, 0.317
RAP (mmHg)	117.94, < 0.001	0.84, 0.466	3.52, 0.018
PCWP (mmHg)	63.04, < 0.001	8.76, < 0.001	1.99, 0.121
CI (L/min/m^2)	8.11, 0.005	32.7, < 0.001	1.38, 0.25
CPO (W)	6.65, 0.01	21.4, < 0.001	1.73, 0.165
CPI (W/m^2)	7.78, 0.006	21.73, <0.001	1.98, 0.122
CPI_{RAP} (W/m^2)	23.72, < 0.001	19.97, < 0.001	3.52, 0.018
PAPI	64.54, < 0.001	0.87, 0.441	1.09, 0.346

CI: Cardiac Index, CPI: Cardiac Power Index, CPI_{RAP}: Cardiac Power Index Right Atrial Pressure corrected, CPO: Cardiac Power Output, HR: Heart Rate, MAP: Mean Arterial Pressure, PAPI: Pulmonary Artery Pulsatility Index, PCWP: Pulmonary Capillary Wedge Pressure, RAP: Right Atrial Pressure, SBP: Systolic Blood Pressure. *F*=*F*-Values, *P*=*P*-values *=interaction.

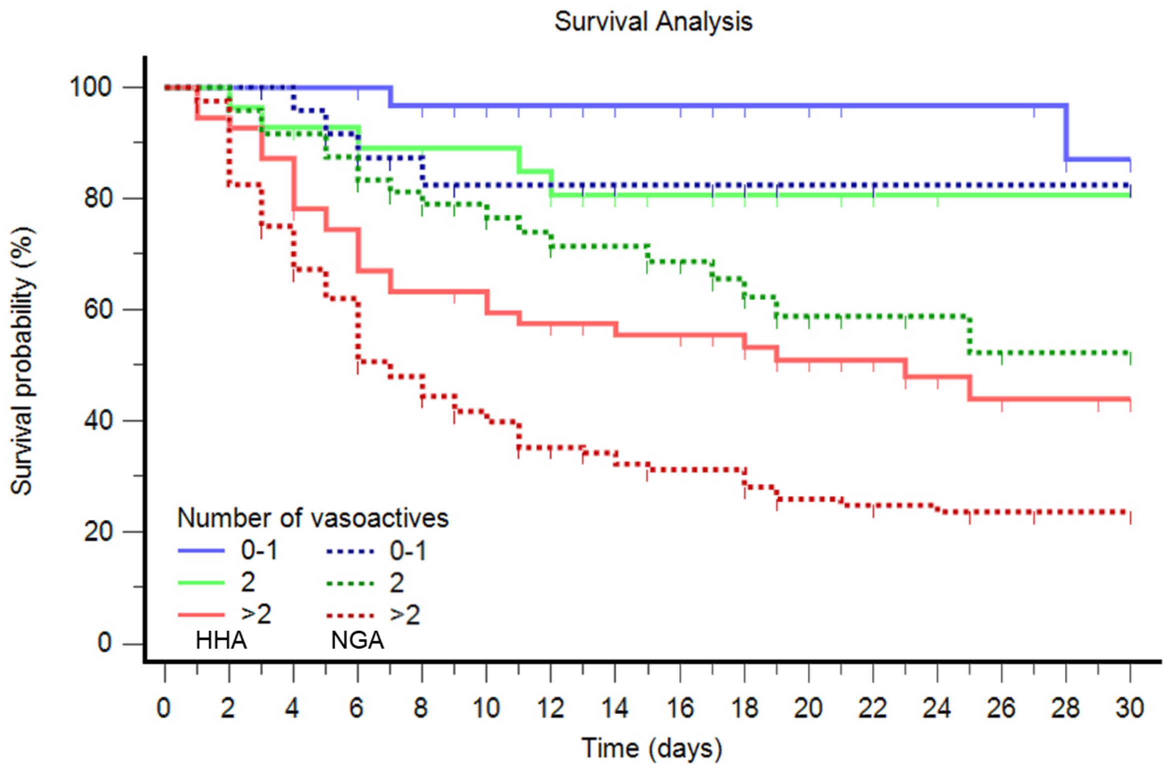

Figure S2. Survival Kaplan-Meir curves for hemodynamic high-achievers (HHA) and non-goal achievers (NGA) groups

The figure, when analyzed, the high-achievers (HHA) had significant differences in the vasoactives groups (Log-rank $P<0.001$) with reduced survival in the restricted mean survival time (RMST) of 3.49 days (-0.33-7.31, P=0.073) in the 2 vasoactive group and 10.96 days (7.43-14.48, $P<0.001$) in the >2 vasoactives. The non-goal-achievers (NGA) had a similar behavior significant difference in the vasoactives groups (Log-rank $P<0.001$) and reduced survival in comparison to 0-1 vasoactives: 4.42 days (-0.51-9.35, $P=0.079$) 2 vasoactives and 13.4 days (9.12-17.69, $P<0.001$) in the >2 vasoactives by RMST.

The hemodynamic goal non-achievers had an increased HR of mortality in comparison to high-achievers HR=2.17 (1.5-3.16, $P<0.001$), when looked by sub-group of vasoactives only >2 vasoactives had an increased HR=1.8 (1.18-2.74, $P=0.006$), in comparison 2 HR=2.08 (0.77-5.61, $P=0.148$), and 0-1 vasoactives HR=2.922 (0.53-15.98, $P=0.216$), although no interaction was noted $Pint=0.957$.

Table S8. Missingness of data per variable and its percentage for Demographic, Clinical, and Laboratory Characteristics of Patients Stratified by Vasoactive Medication Levels in Acute Myocardial Infarction-Related Cardiogenic Shock.

Variables	Complete data by variable
Gender (%)	309 (100%)
Age (years)	309 (100%)
BMI (kg/m2)	309 (100%)
Hypertension (%)	309 (100%)
DM2 (%)	309 (100%)
Previous HF (%)	309 (100%)
Previous AMI (%)	309 (100%)
Previous PCI (%)	309 (100%)
Previous CABG (%)	309 (100%)
Smoking history (%)	309 (100%)
OHCA (%)	309 (100%)
Type of AMI (%)	309 (100%)
Killip–Kimball (%)	309 (100%)
Type of primary reperfusion (%)	309 (100%)
LVEF (%)	309 (100%)
Hemoglobin (g/dL)	309 (100%)
Leucocytes (cells/mm3)	309 (100%)
Neutrophils (%)	307 (99%)
Platelets (cells/mm3)	309 (100%)
Glucose (mg/dL)	309 (100%)
BUN (mg/dL)	309 (100%)
Creatinine (mg/dL)	309 (100%)
eGFR (ml/min/1.73m2)	309 (100%)
Sodium (mEq/L)	309 (100%)
Potassium (mEq/L)	307 (99%)
Chloride (mEq/L)	305 (99%)
Albumin (g/L)	287 (93%)
AST (U/L)	300 (97%)
ALT (U/L)	289 (94%)
LDH (U/L)	289 (94%)
C-reactive protein (mg/L)	283 (92%)
Maximum creatinine (mg/dL)	309 (100%)
Maximum AST (mg/dL)	301 (97%)
Maximum ALT (U/L)	289 (94%)
Minimum PAFI (paO2/FiO2%)	309 (100%)
Maximum 24h Lactate (mmol/L)	302 (98%)
Minimum 24h Excess base	302 (98%)
Minimum 24h pH	302 (98%)
Mechanical circulatory support (%)	309 (100%)
Angiography (%)	309 (100%)
Number of vessels affected > 50% (%)	309 (100%)
Total PCI (%)	309 (100%)
Dobutamine (%)	309 (100%)
Levosimendan (%)	309 (100%)
Norepinephrine (%)	309 (100%)
Vasopressin (%)	309 (100%)
Hemodialysis (%)	309 (100%)
Mechanical ventilation (%)	309 (100%)
Total stay length (days)	309 (100%)
SCAI	309 (100%)
MODS score	309 (100%)
Multiorgan failure (%)	309 (100%)
Number of organ failures (%)	309 (100%)
AKI (%)	309 (100%)
AKIN stages (%)	309 (100%)
In-hospital mortality (%)	309 (100%)

Table S9. Missingness of data per variable and its percentage for the hemodynamic data in Acute Myocardial Infarction-Related Cardiogenic Shock.

	0 hours	6 hours	12 hours	24 hours
HR (bpm)	309 (100%)	286 (92%)	269 (87%)	245 (79%)
SBP (mmHg)	309 (100%)	286 (92%)	269 (87%)	245 (79%)
MAP (mmHg)	309 (100%)	286 (92%)	269 (87%)	245 (79%)
RAP (mmHg)	307 (99%)	286 (92%)	268 (86%)	245 (79%)
PCWP (mmHg)	307 (99%)	286 (92%)	269 (87%)	245 (79%)
CI (L/min/m2)	307 (99%)	285 (92%)	269 (87%)	245 (79%)
CPO (W)	307 (99%)	285 (92%)	269 (87%)	245 (79%)
CPI (W/m2)	307 (99%)	285 (92%)	269 (87%)	245 (79%)
CPIRAP (W/m2)	307 (99%)	285 (92%)	268 (86%)	245 (79%)
PAPI	304 (98%)	281 (90%)	267 (86%)	238 (77%)

Directed acyclic graph for the multivariable assessment (Above and below rotated for easy eye tracking Figure S3) DGA view of relevant clinical variables and it relationship with the number of vasoactives and mortality

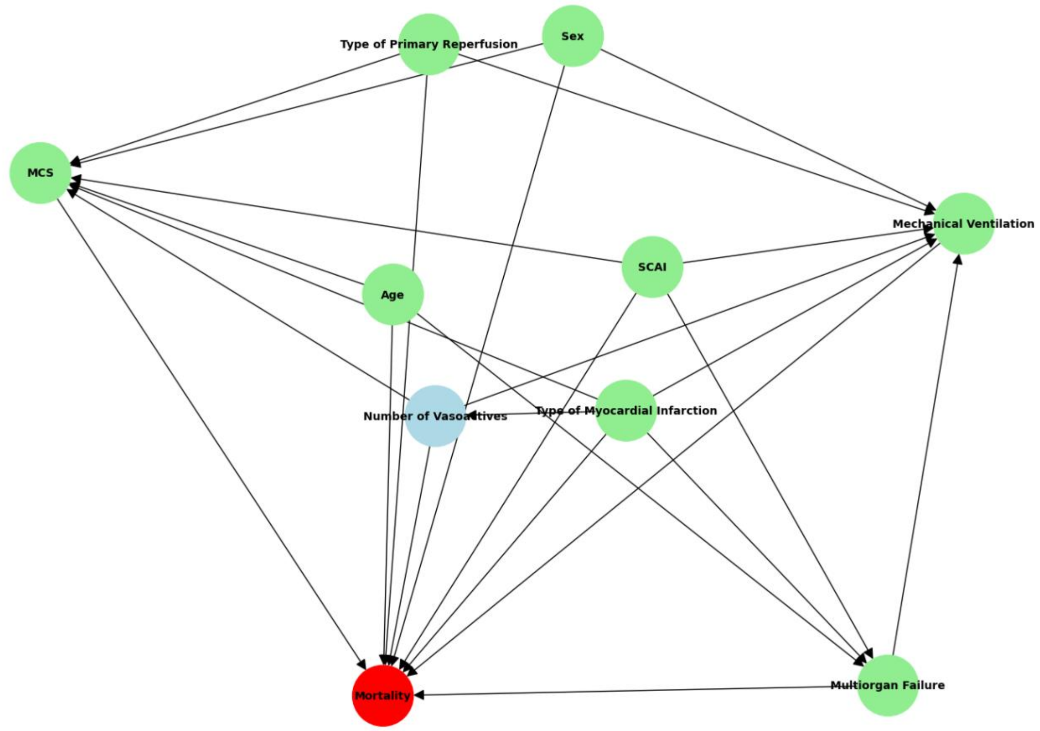

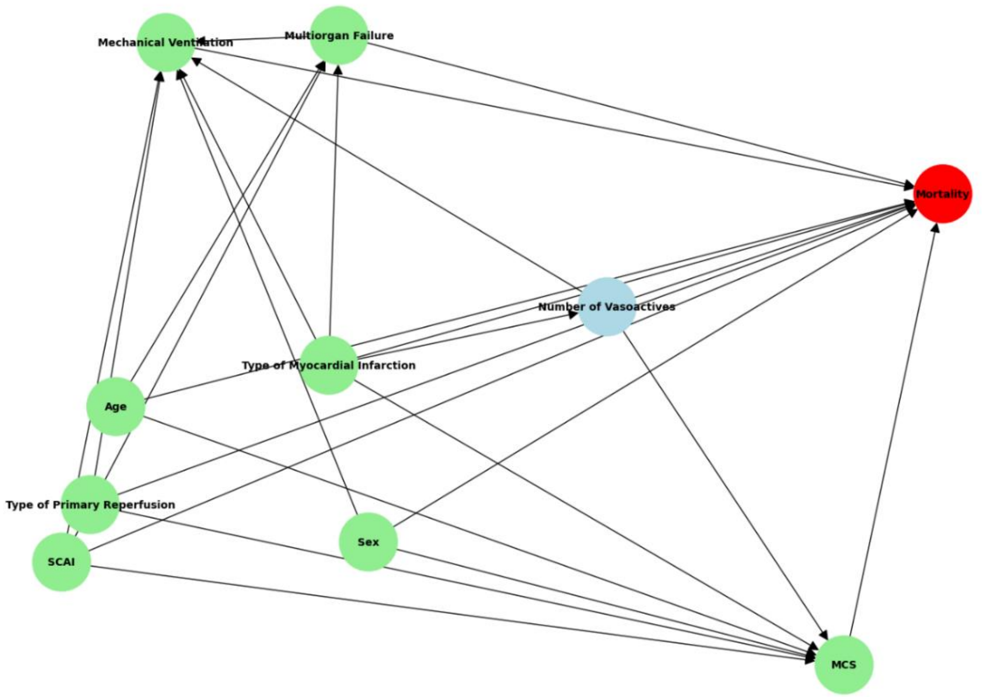

The directed acyclic graph (DAG) provides a comprehensive visualization of the complex interactions between demographic, clinical, and treatment-related factors influencing patient mortality in critical cardiovascular conditions. At its core, the DAG highlights the role of key variables such as age, sex, clinical status markers, and therapeutic interventions.

Demographic factors like age and sex play foundational roles in determining outcomes. Age, for instance, directly impacts the risk of mortality while also contributing to intermediate factors such as multiorgan failure and the need for mechanical circulatory support (MCS). Sex differences, on the other hand, may influence disease severity and the utilization of specific interventions, such as mechanical ventilation.

Clinical status markers, including multiorgan failure and the number of vasoactive agents required, are critical indicators of disease severity. These variables predict mortality and mediate the effects of other upstream factors, such as the severity index captured by the SCAI Shock Classification. This classification system provides a structured framework for assessing patient risk and guiding the escalation of care.

Treatment-related variables, such as the type of primary reperfusion and the type of myocardial infarction, are central to the causal framework. These factors influence clinical trajectories by determining the severity of organ dysfunction and the need for advanced supportive measures like MCS or mechanical ventilation. For example, the type of myocardial infarction—whether STEMI or NSTEMI—shapes therapeutic decisions and downstream outcomes by mediating its effects through organ failure and the use of vasoactive agents.

At the heart of the DAG lies mortality, the ultimate endpoint of interest. Mortality is affected by direct and indirect pathways, with upstream variables influencing it through a network of mediators. This structured representation of causal relationships offers significant insights for clinical research and practice, helping to identify key mediators, potential confounders, and avenues for targeted interventions.

Logistic regression modeling

With the selected variables, we made a significant model regression with X_2=105.14 P<0.001 with a R^2=0.39 and a goodness of fit for the Hosmer-Lemeshow of X_2=11.58 P=0.171.

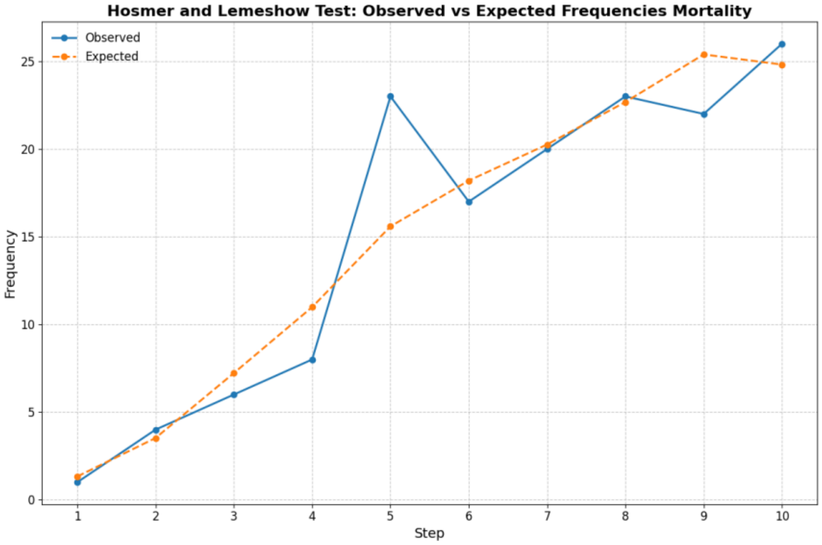

Table S10. Multivariate assessment with mortality:

Variable	B	SE	Wald	gl	Sig.	Exp(B)	95% CI para EXP(B)	
							Inferior	Superior
Sex	-0.097	0.376	0.067	1	0.796	0.907	0.434	1.896
Age	0.045	0.014	10.239	1	0.001	1.046	1.018	1.075
Type of myocardial infarction	-1.151	0.593	3.767	1	0.052	0.316	0.099	1.011
Type of reperfusion			4.266	2	0.119			
Pharmacoinvasive	0.003	0.628	0	1	0.997	1.003	0.293	3.43
pPCI	-0.643	0.317	4.109	1	0.043	0.526	0.283	0.979
MCS	0.248	0.302	0.672	1	0.412	1.281	0.709	2.316
SCAI			1.735	2	0.42			
SCAI D	0.738	0.572	1.667	1	0.197	2.091	0.682	6.412
SCAI E	0.729	0.583	1.562	1	0.211	2.073	0.661	6.501
Mechanical ventilation	1.13	0.355	10.125	1	0.001	3.095	1.543	6.206
MOF	0.103	0.309	0.111	1	0.739	1.109	0.604	2.033
Number of vasoactives (Ref=0-1)			24.152	2	<0.001			
2	0.845	0.514	2.701	1	0.1	2.329	0.85	6.38
>2	2.047	0.492	17.294	1	<0.001	7.743	2.951	20.316
Constant	-4.369	1.075	16.515	1	0	0.013		

Schoenfeld Residuals Test:

**Proportionality hazard test**	*P*-value
Global	0.224
Global adjusted	0.245
High goal-achievers	0.121
High goal-achievers adjusted	0.316
SBP ≥90 (mmHg)	0.228
SBP ≥90 (mmHg) adjusted	0.193
MAP ≥65 (mmHg)	0.304
MAP ≥65 (mmHg) adjusted	0.329
PCWP<18 (mmHg)	**0.029***
PCWP<18 (mmHg) adjusted	0.104
CI ≥2.2 (L/min/m2)	0.616
CI ≥2.2 (L/min/m2) adjusted	0.863
CPO≥0.6 (W)	0.567
CPO≥0.6 (W) adjusted	0.692
CPI ≥0.32 (W/m2)	0.401
CPI ≥0.32 (W/m2) adjusted	0.576
CPIrap ≥0.28 (W/m2)	0.602
CPIrap ≥0.28 (W/m2) adjusted	0.74
PAPI ≥1	0.138
PAPI ≥1 adjusted	0.597
Dobutamine	0.174
Dobutamine adjusted	0.163
Levosimendan	**0.024**
Levosimendan adjusted	0.068
Vasopressin	**0.022**
Vasopressin adjusted	0.114
Norepinephrine	0.202
Norepinephrine adjusted	0.197
HHA-Dobutamine	0.175
HHA-Dobutamine adjusted	0.163
HHA-Levosimendan	**0.025**
HHA-Levosimendan adjusted	0.068
HHA-Vasopressin	**0.022**
HHA-Vasopressin adjusted	0.114
HHA-Norepinephrine	0.202
HHA-Norepinephrine adjusted	0.197

*For PCWP, levosimendan and vasopressin time varying analysis was carry out 1, 71 15 and 30 days

Tables S11. Time varying analysis was carry out 1, 71 15 and 30 days for (A) PCWP, (B) Levosimendan and vasopressin and (C) HHA-levosimendan and HHA-vasopressin

(A) HR (time)	Number of vasoactives=2	*P*-value	Number of vasoactives>2	*P*-value
**PCWP HR_{Day1}**	1.66 (0.8-3.45)	0.171	5.57 (3.1-10.02)	<0.001
**PCWP HR_{Day7}**	1.36 (0.7-2.64)	0.365	3.99 (2.3-6.92)	<0.001
**PCWP HR_{Day15}**	1.04 (0.55-1.96)	0.911	2.56 (1.49-4.39)	0.001
**PCWP HR_{Day30}**	0.63 (0.28-1.37)	0.242	1.11 (0.59-2.09)	0.747

(B) HR (Time)	Levosimendan HR (95%CI)	P-value	Vasopressin HR (95%CI)	P-value
**Day 1**	2.67 (1.82-3.91)	<0.001	8.77 (6.04-12.75)	<0.001
**Day 7**	2.0 (1.44-2.79)	<0.001	5.84 (4.16-8.19)	<0.001
**Day 15**	1.36 (0.99-1.87)	0.055	3.39 (2.44-4.71)	<0.001
**Day 30**	0.66 (0.42-1.03)	0.069	1.23 (0.8-1.87)	0.350

(C) HR (Time)	HHA-Levosimendan HR (95%CI)	P-value	HHA-Vasopressin HR (95%CI)	P-value
**Day 1**	2.62 (1.22-5.65)	0.014	7.61 (3.88-14.94)	<0.001
**Day 7**	2.08 (1.06-4.08)	0.033	5.28 (2.87-9.72)	<0.001
**Day 15**	1.53 (0.82-2.88)	0.185	3.24 (1.8-5.84)	<0.001
**Day 30**	0.86 (0.38-1.96)	0.722	1.3 (0.61-2.74)	0.499

Table S12 & Figure S4. Regression Analysis of Vasoactive Agents and Their Interaction with Time on PCWP

PCWP	coef	exp(coef)	se(coef)	Coef lower 95%	coef upper 95%	exp(coef) lower 95%	exp(coef) upper 95%	p	-log2(p)
**Number of vasoactives=2**	0.54	1.72	0.38	-0.20	1.29	0.82	3.62	0.15	2.71
**Number of vasoactives>2**	1.77	5.89	0.30	1.18	2.37	3.25	10.67	<0.005	27.49
**Number of vasoactives=2*time**	-0.03	0.97	0.01	-0.06	-0.01	0.94	0.99	0.02	5.67
**Number of vasoactives>2*time**	-0.06	0.95	0.01	-0.08	-0.04	0.93	0.96	<0.005	25.50

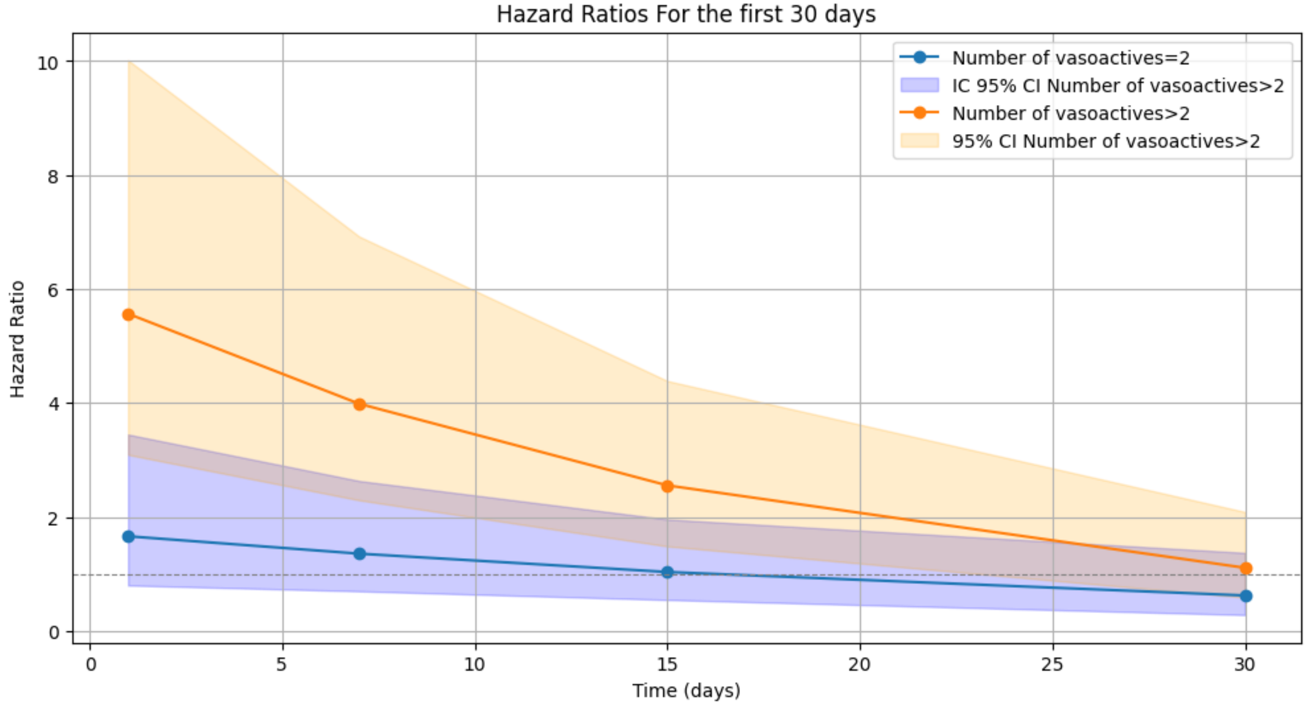

Table S13 & Figure S5. Regression Analysis of Vasoactive Agents and Their Interaction with Time on Levosimendan

Levosimendan	coef	exp(coef)	se(coef)	coef lower 95%	coef upper 95%	exp(coef) lower 95%	exp(coef) upper 95%	p	-log2(p)
**Levosimendan**	1.03	2.80	0.20	0.64	1.42	1.89	4.15	<0.005	21.75
**Levosimendan*time**	-0.05	0.95	0.01	-0.07	-0.03	0.94	0.97	<0.005	21.44

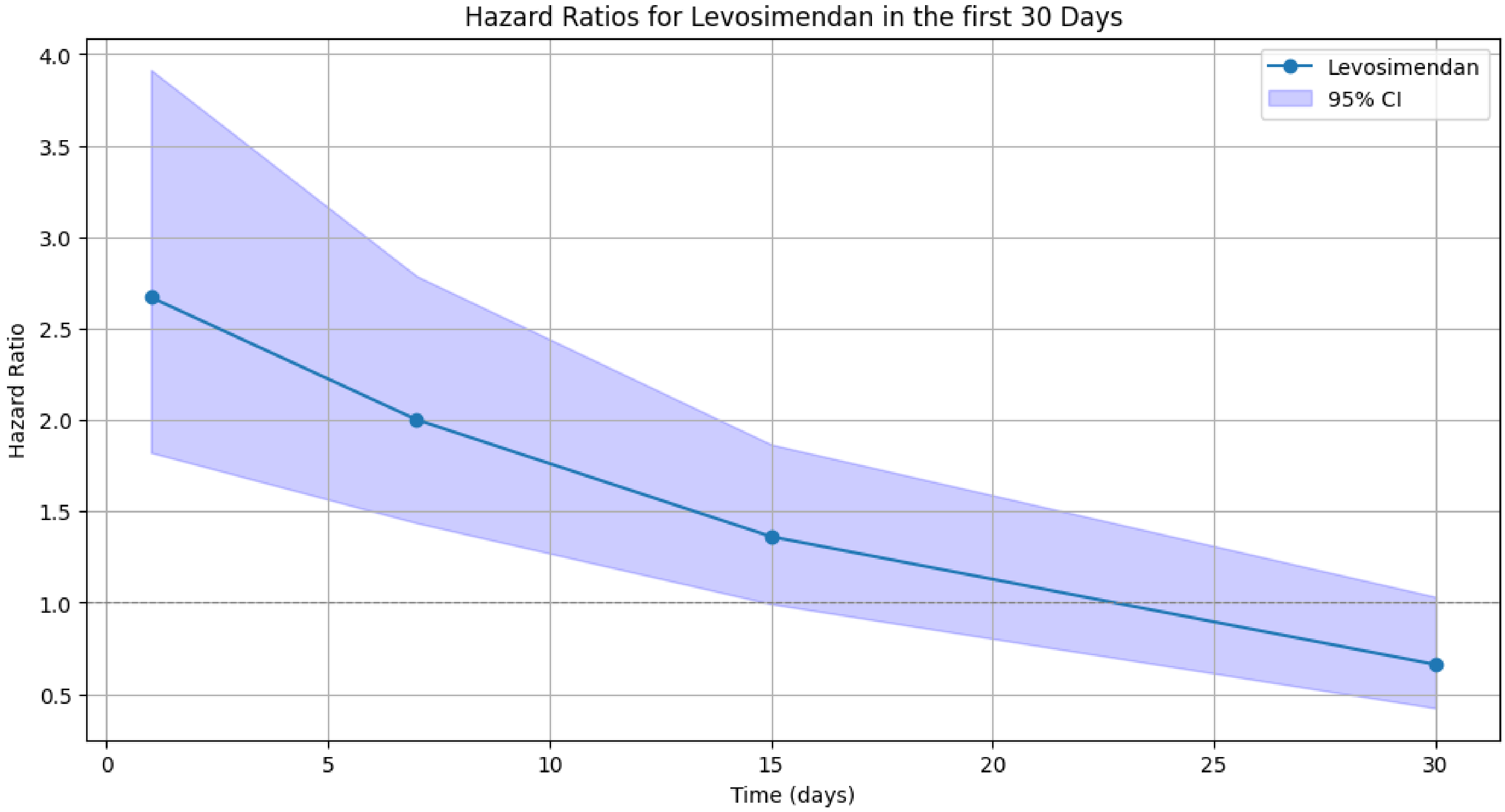

Table S14 & Figure S6. Regression Analysis of Vasoactive Agents and Their Interaction with Time on Vasopressin

	coef	exp(coef)	se(coef)	coef lower 95%	coef upper 95%	exp(coef) lower 95%	exp(coef) upper 95%	p	-log2(p)
**Vasopressin**	2.24	9.39	0.19	1.86	2.62	6.41	13.75	<0.005	99.40
**Vasopressin*time**	-0.07	0.93	0.01	-0.08	-0.05	0.92	0.95	<0.005	56.08

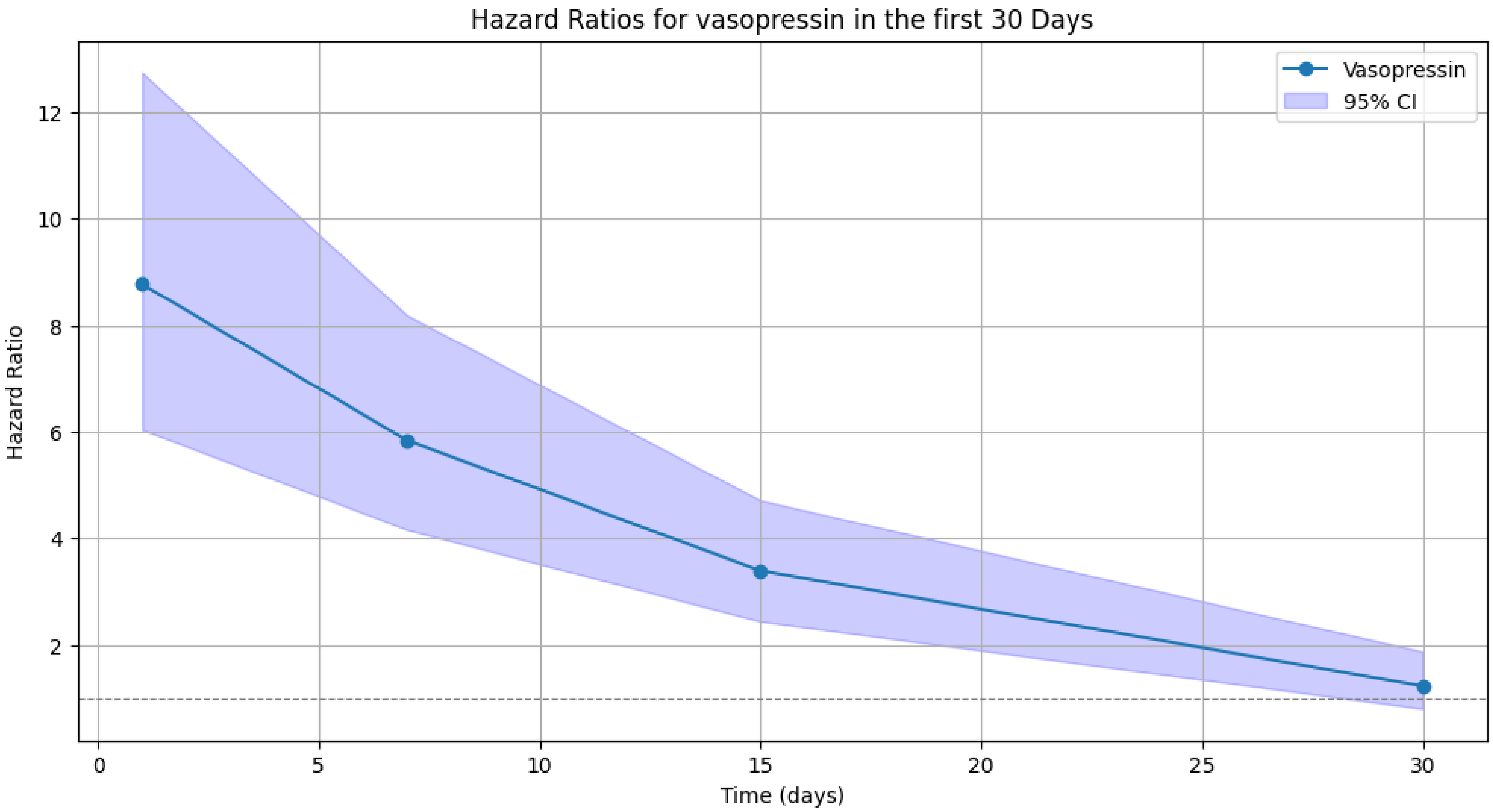

Table S15 & Figure S7. Regression Analysis of Vasoactive Agents and Their Interaction with Time on HHA-Levosimendan

HHA-Levosimendan	coef	exp(coef)	se(coef)	coef lower 95%	coef upper 95%	exp(coef) lower 95%	exp(coef) upper 95%	p	-log2(p)
**HHA-Levosimendan**	1.00	2.73	0.40	0.22	1.79	1.24	5.98	0.01	6.33
**HHA-Levosimendan*time**	-0.04	0.96	0.02	-0.07	-0.01	0.93	0.99	0.02	5.38

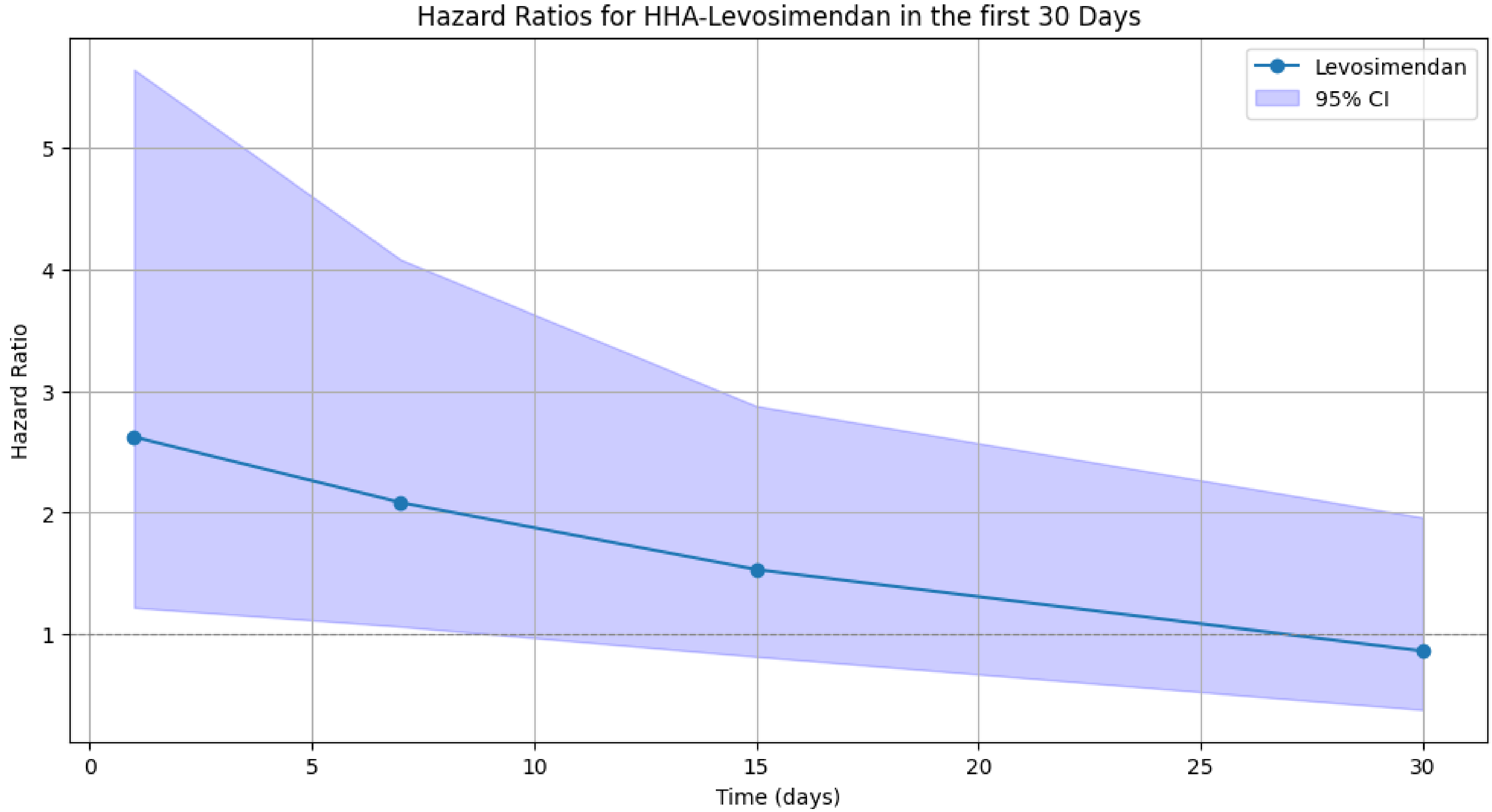

Table S16 & Figure S8. Regression Analysis of Vasoactive Agents and Their Interaction with Time on HHA-Vassopresin

HHA-Vasopressin	coef	exp(coef)	se(coef)	coef lower 95%	coef upper 95%	exp(coef) lower 95%	exp(coef) upper 95%	p	-log2(p)
**HHA-Vasopressin**	2.09	8.09	0.35	1.40	2.78	4.07	16.10	<0.005	28.55
**HHA-Vasopressin*time**	-0.06	0.94	0.01	-0.09	-0.03	0.92	0.97	<0.005	16.27

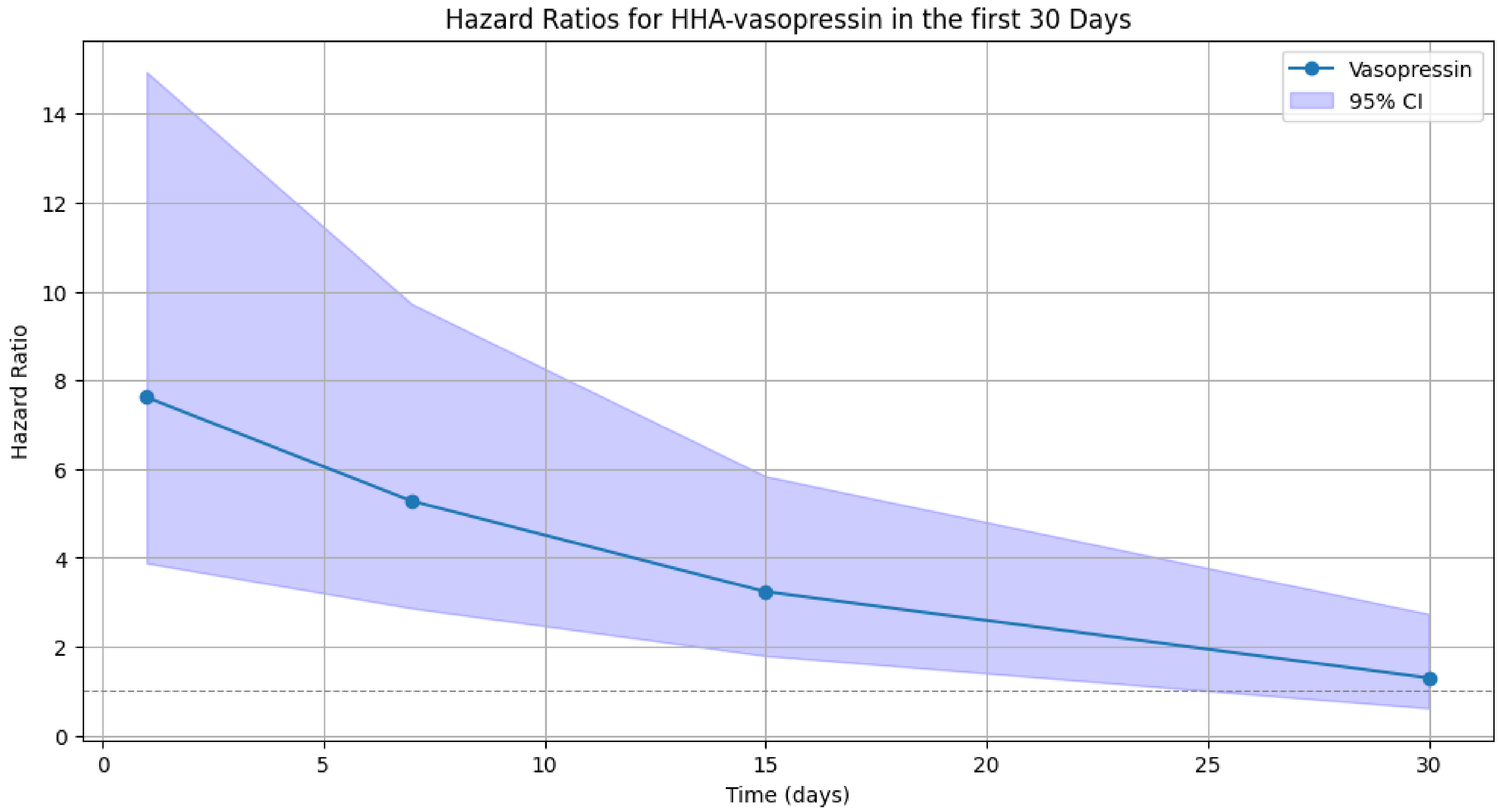

P-interaction Test:

Variable	P_{int}-value
High goal-achievers	0.975
High goal-achievers adjusted	0.79
SBP ≥90 (mmHg)	0.142
SBP ≥90 (mmHg) adjusted	0.315
MAP ≥65 (mmHg)	**0.048**
MAP ≥65 (mmHg) adjusted	0.22
PCWP<18 (mmHg)	0.796
PCWP<18 (mmHg) adjusted	0.944
CI ≥2.2 (L/min/m2)	0.492
CI ≥2.2 (L/min/m2) adjusted	0.704
CPO≥0.6 (W)	0.976
CPO≥0.6 (W) adjusted	0.913
CPI ≥0.32 (W/m2)	0.082
CPI ≥0.32 (W/m2) adjusted	0.191
CPIrap ≥0.28 (W/m2)	0.622
CPIrap ≥0.28 (W/m2) adjusted	0.621
PAPI ≥1	0.496
PAPI ≥1 adjusted	0.653
HHA-Dobutamine	0.517
HHA-Dobutamine adjusted	0.853
HHA-Levosimendan	0.46
HHA-Levosimendan adjusted	0.364
HHA-Vasopressin	0.779
HHA-Vasopressin adjusted	0.85
HHA-Norepinephrine	0.142
HHA-Norepinephrine adjusted	0.144

*HR for MAP<65 mmHg unadjusted for number of vasoactives=2: 2.69 (1.07-6.79) *P*=0.036; and for >2: 8.25 (3.61-18.84) *P*<0.001.

Sensitivity test

We add considering 'number of vasoactive drugs as a numerical data and we consider restricted cubic splines to model the non-linear relationship with the outcome. Based on the analysis of residuals and the comparison between the linear and non-linear models using restricted cubic splines, we observed no significant improvement in model performance when employing non-linear transformations. The likelihood ratio test yielded a P=0.098, which indicates that the restricted cubic splines did not provide a statistically significant improvement over the simpler linear model. Furthermore, the visual inspection of residuals did not reveal any systematic patterns or substantial deviations that would suggest non-linearity. And the Box-Tidwell log-odds P=0.233 confidently conclude that a linear model is appropriate. Given these findings, a linear relationship between the number of vasoactive drugs and mortality was deemed appropriate and sufficiently robust for our analysis. (Figures S9 and S10)

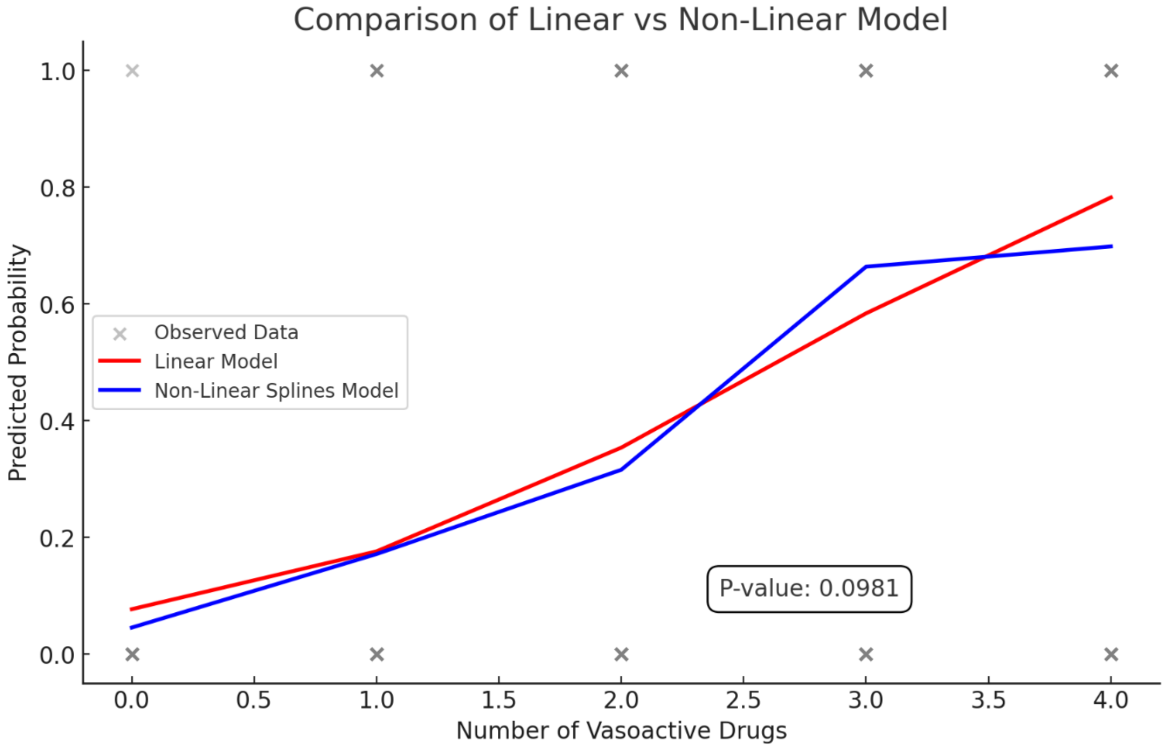

Figure S9. Comparison between linear vs non-linear model in logistic regresion

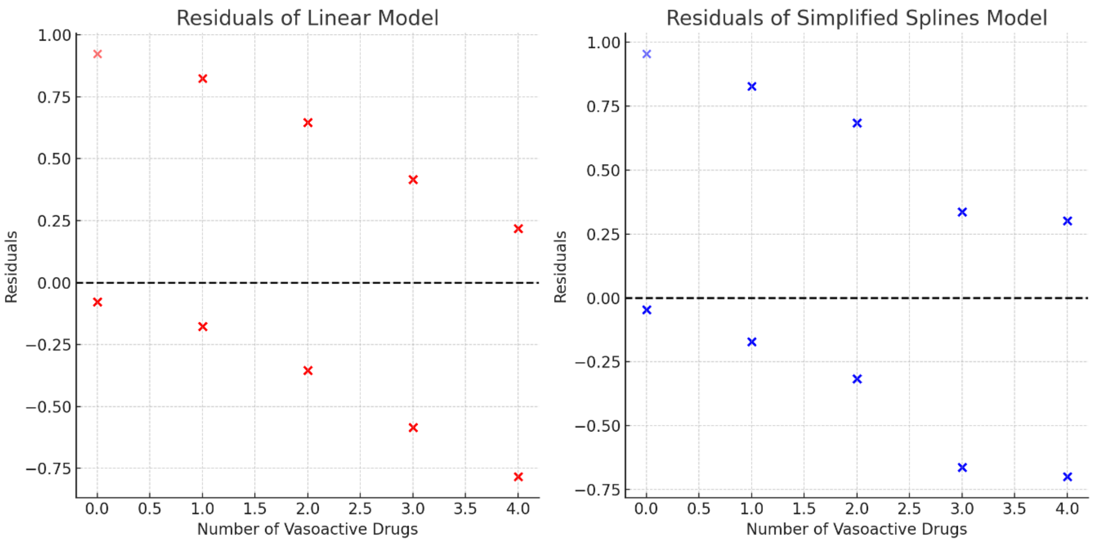

Figure S10. Comparison between residuals linear vs non-linear model in logistic regresion

The comparison between the linear Cox model and the restricted cubic spline model showed no significant improvement in the model fit when using splines. The likelihood ratio test yielded a p-value of 1.00, indicating no evidence of non-linearity in the relationship between the number of vasoactive drugs and the hazard ratio. Additionally, the predicted hazards from both models were visually similar, and the spline model introduced unnecessary complexity without enhancing the interpretability or predictive performance. Additionally, the evaluation of Martingale residuals against the number of vasoactive drugs reveals no systematic deviation or non-random patterns that would suggest a non-linear relationship. The residuals are scattered around zero without strong evidence of curvature, further supporting the appropriateness of a linear relationship. Therefore, the linear Cox model was selected as it provides a more parsimonious and robust representation of the data. (Figure S11 ands S12)

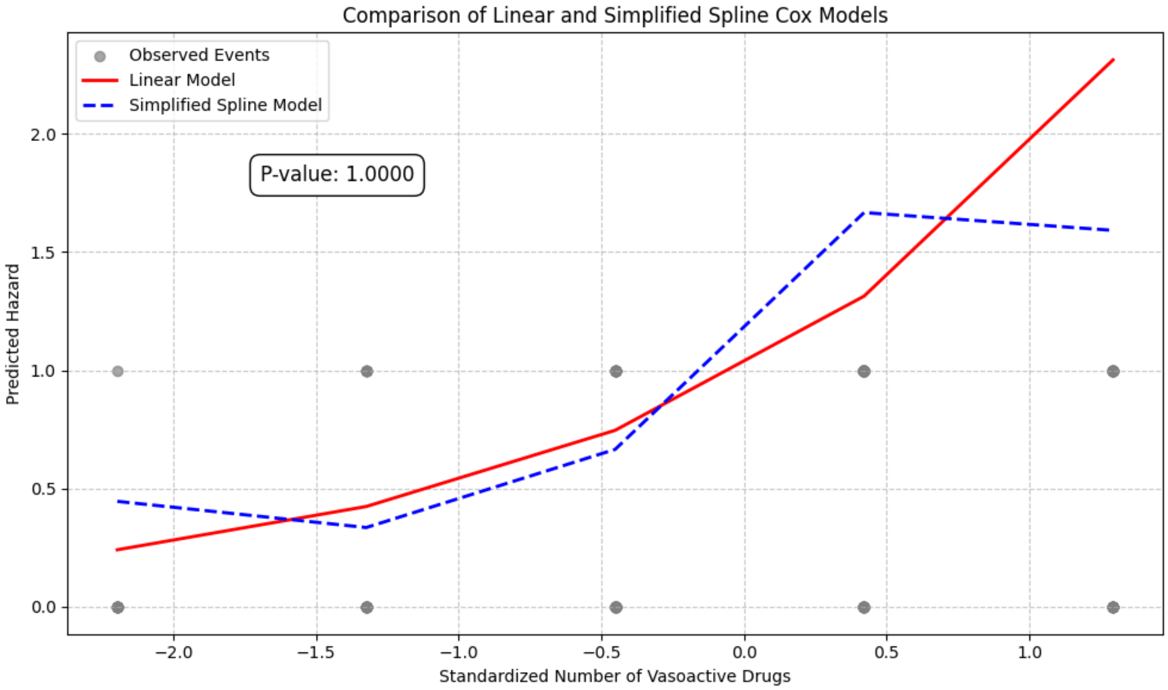

Figure S11. Comparison between linear vs non-linear model in Cox regression

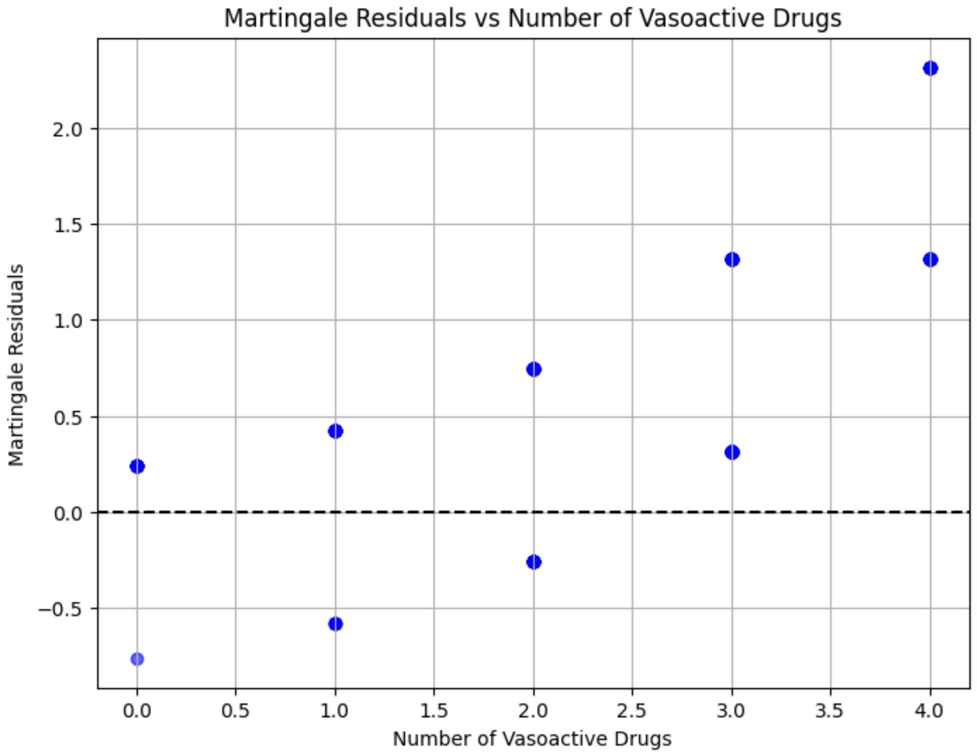

Figure S12.Comparison residuals between in non-linear model in Cox regression

Comparison of Sensitivity vs. Original Analysis

Both the sensitivity and original analyses showed consistent trends across key parameters, demonstrating robust findings. Lower systolic blood pressure (▲10 mmHg) and mean arterial pressure (▲10 mmHg) were strongly associated with reduced odds of adverse outcomes at all time points, with nearly identical odds ratios and significance levels in both analyses. Similarly, lower cardiac power output and cardiac power index emerged as protective factors at later time points, with consistent results in magnitude and significance. These findings highlight the stability of these parameters as predictors of improved outcomes.

The use of vasoactive medications showed a significant number of vasoactives-response relationships in both analyses, with each increase in vasoactives markedly increasing the odds of adverse outcomes. Parameters such as cardiac index and pulmonary artery pulsatility index demonstrated no significant associations in either analysis, underscoring their limited predictive value in this context. While elevated right atrial pressure and pulmonary capillary wedge pressure showed associations with worse outcomes at certain time points, these findings were less consistent across analyses, reflecting potential variability in their predictive value.

Overall, the sensitivity and original analyses were largely concordant, with minor differences in effect size and statistical significance attributable to modeling variations but only seen in the unadjusted models (marked with yellow in Table SXXX). The consistency of trends across both analyses underscores the robustness of the relationships between key hemodynamic parameters, vasoactive use, and patient outcomes, providing confidence in the validity of the findings.

The sensitivity analysis also consistently showed stronger associations for all variables compared to the original analysis, with higher hazard ratios (HRs) and lower *p*-values, reflecting amplified effects

of increasing the numbers of vasoactives use. Hemodynamic goals, such as achieving ≥5/6 goals (HHA), SBP ≥90 mmHg, and MAP ≥65 mmHg, exhibited more pronounced effects in the sensitivity analysis but remained significant in the original analysis. Parameters like cardiac index (CI ≥2.2 L/min/m²) and cardiac power output (CPO ≥0.6 W) followed similar trends, with significance in both analyses but higher HRs in the sensitivity analysis. While PCWP <18 mmHg had proportionality in the sensitivity analysis it had time dependent fashion in the original analysis. Interaction tests and Schoenfeld residuals confirmed proportional hazards across both analyses in other variables, with the sensitivity analysis generally amplifying associations while the original analysis offered more conservative estimates. (table SXXX)

OR (95 %CI; *P*-value)	0 hours	6 hours	12 hours	24 hours
Systolic Blood Pressure (▲10 mmHg)	0.82 (0.71-0.94; 0.006)	0.83 (0.71-0.96; 0.017)	0.81 (0.68-0.96; 0.017)	0.69 (0.57-0.81; <0.001)
+1 vasoactives	2.44 (1.87-3.19; <0.001)	2.48 (1.9-3.24; <0.001)	2.44 (1.86-3.19; <0.001)	2.42 (1.84-3.18; <0.001)
Mean Arterial Pressure (▲10 mmHg)	0.78 (0.64-0.94; 0.01)	0.75 (0.6-0.91; 0.006)	0.77 (0.61-0.97; 0.027)	0.61 (0.48-0.78; <0.001)
+1 vasoactives	2.45 (1.85-3.19; <0.001)	2.49 (1.19-3.25; <0.001)	2.45 (1.88-3.2; <0.001)	2.41 (1.84-3.16; <0.001)
Cardiac index (▲0.5 L/m^2)	0.98 (0.83-1.14;0.756)	0.88 (0.75-1.02; 0.089)	0.91 (0.78-1.06; 0.201)	0.89 (0.76-1.05; 0.16)
+1 vasoactives	2.54 (1.94-3.33; <0.001)	2.47 (1.89-3.23; <0.001)	2.52 (1.93-3.28; <0.001)	2.51 (1.92-3.27; <0.001)
Cardiac power output (▲0.1 W)	0.93 (0.85-1.01; 0.095)	0.9 (0.83-0.98; 0.013)	0.91 (0.83-0.99; 0.036)	0.87 (0.8-0.95; 0.003)
+1 vasoactives	2.42 (1.85-3.17; <0.001)	2.41 (1.84-3.15; <0.001)	2.44 (1.87-3.19; <0.001)	2.4 (1.83-3.13; <0.001)
Cardiac power index (▲0.1 W/m^2)	0.89 (0.75-1.04; 0.136)	0.84 (0.72-0.97; 0.023)	0.85 (0.72-1.0; 0.046)	0.78 (0.65-0.92; 0.003)
+1 vasoactives	2.43 (1.85-3.19; <0.001)	2.42 (1.85-3.17; <0.001)	2.45 (1.88-3.2; <0.001)	2.4 (1.84-3.14; <0.001)
$CPI_{(RAP)}$ (▲0.1 W/m^2)	0.83 (0.69-0.99; 0.042)	0.78 (0.65-0.92; 0.005)	0.78 (0.64-0.94; 0.009)	0.71 (0.58-0.86; 0.001)
+1 vasoactives	2.38 (1.81-3.12; <0.001)	2.36 (1.8-3.1; <0.001)	2.38 (1.81-3.12; <0.001)	2.33 (1.78-3.06; <0.001)
PAPI (▲ 1 unit)	1.01 (0.84-1.22; 0.906)	0.99 (0.78-1.27; 0.914)	0.81 (0.62-1.04; 0.096)	0.9 (0.68-1.17; 0.419)
+1 vasoactives	2.57 (1.97-3.36; <0.001)	2.55 (1.95-3.35; <0.001)	2.47 (1.89-3.23; <0.001)	2.52 (1.93-3.29; <0.001)
RAP (▲1 mmHg)	1.03 (0.99-1.07; 0.124)	1.07 (1.02-1.13; 0.005)	1.07 (1.02-1.12; 0.01)	1.04 (0.99-1.09; 0.145)
+1 vasoactives	2.51 (1.92-3.28; <0.001)	2.44 (1.86-3.21; <0.001)	2.4 (1.83-3.14; <0.001)	2.48 (1.89-3.24; <0.001)
PCWP (▲1 mmHg)	1.04 (1.0-1.07; 0.055)	1.07 (1.02-1.11; 0.004)	1.04 (1.0-1.08; 0.072)	1.05 (1.0-1.09; 0.056)
+1 vasoactives	2.51 (1.92-3.26; <0.001)	2.52 (1.93-3.29; <0.001)	2.54 (1.95-3.31; <0.001)	2.49 (1.91-3.25; <0.001)

Table S17: Sensitivity test for Unadjusted Bivariate Relationship Between Hemodynamic Parameters and Vasoactive Medications at Different Time Intervals.

OR (95 %CI; *P*-value)	0 hours	6 hours	12 hours	24 hours
Systolic Blood Pressure (▲10 mmHg)	0.83 (0.71-0.95; 0.011)	0.81 (0.68-0.96; 0.032)	0.79 (0.65-0.94; 0.011)	0.68 (0.56-0.81; <0.001)
+1 vasoactives	1.89 (1.39-2.59; <0.001)	1.9 (1.39-2.59; <0.001)	1.83 (1.33-2.51; <0.001)	1.84 (1.33-2.54; <0.001)
Mean Arterial Pressure (▲10 mmHg)	0.79 (0.64-0.97; 0.025)	0.74 (0.59-0.94; 0.023)	0.77 (0.6-0.99; 0.037)	0.65 (0.5-0.83; 0.001)
+1 vasoactives	1.89 (1.39-2.58; <0.001)	1.93 (1.42-2.63; <0.001)	1.88 (1.37-2.56; <0.001)	1.88 (1.37-2.58; <0.001)
Cardiac index (▲0.5 L/m^2)	1.01 (0.85-1.2; 0.932)	0.91 (0.76-1.07; 0.263)	0.95 (0.8-1.12; 0.56)	0.94 (0.79-1.12; 0.477)
+1 vasoactives	1.96 (1.44-2.68; <0.001)	1.92 (1.41-2.62; <0.001)	1.95 (1.43-2.65; <0.001)	1.94 (1.42-2.34; <0.001)
Cardiac power output (▲0.1 W)	0.95 (0.86-1.04; 0.249)	0.92 (0.83-1.01; 0.08)	0.93 (0.85-1.03; 0.166)	0.89 (0.81-0.99; 0.025)
+1 vasoactives	1.89 (1.38-2.58; <0.001)	1.88 (1.38-2.57; <0.001)	1.91 (1.4-2.6; <0.001)	1.87 (1.37-2.55; <0.001)
Cardiac power index (▲0.1 W/m^2)	0.91 (0.77-1.08; 0.306)	0.87 (0.73-1.03; 0.122)	0.89 (0.74-1.06; 0.207)	0.82 (0.68-0.99; 0.034)
+1 vasoactives	1.9 (1.39-2.59; <0.001)	1.89 (1.39-2.59; <0.001)	1.92 (1.4-2.61; <0.001)	1.88 (1.38-2.56; <0.001)
$CPI_{(RAP)}$ (▲0.1 W/m^2)	0.86 (0.7-1.04; 0.137)	0.8 (0.65-0.97; 0.034)	0.83 (0.67-1.01; 0.069)	0.76 (0.6-0.94; 0.01)
+1 vasoactives	1.86 (1.36-2.55; <0.001)	1.85 (1.35-2.53; <0.001)	1.88 (1.37-2.57; <0.001)	1.83 (1.34-2.5; <0.001)
PAPI (▲ 1 unit)	1.02 (0.83-1.27; 0.826)	0.98 (0.74-1.29; 0.869)	0.83 (0.63-1.08; 0.169)	0.88 (0.65-1.19; 0.398)
+1 vasoactives	1.97 (1.44-2.68; <0.001)	1.95 (1.42-2.67; <0.001)	1.9 (1.39-2.6; <0.001)	1.92 (1.4-2.62; <0.001)
RAP (▲1 mmHg)	1.03 (0.98-1.07; 0.234)	1.08 (1.02-1.14; 0.006)	1.06 (1.0-1.11; 0.036)	1.04 (0.99-1.1; 0.141)
+1 vasoactives	1.94 (1.42-2.64; <0.001)	1.85 (1.35-2.54; <0.001)	1.88 (1.37-2.57; <0.001)	1.88 (1.37-2.57; <0.001)
PCWP (▲1 mmHg)	1.02 (0.98-1.06; 0.316)	1.06 (1.01-1.11; 0.012)	1.03 (0.99-1.08; 0.133)	1.04 (0.99-1.09; 0.144)
+1 vasoactives	1.95 (1.43-2.65; <0.001)	1.95 (1.43-2.67; <0.001)	1.95 (1.43-2.66; <0.001)	1.94 (1.42-2.64; <0.001)

Table S18: Sensitivity test for **Unadjusted Bivariate Relationship Between Hemodynamic Parameters and Vasoactive Medications at Different Time Intervals.**

Table S18: Sensitivity test for Hazar Ratio Relationship Between Hemodynamic Goal Achievers and Vasoactive Medications

Variable	HR	P- Value	Schoenfeld P	P-int	HRa	P- Value	Schoenfeld P	Pint
**+1 Vasoactives**	1.74 (1.48-2.05)	<0.001	0.533		1.43 (1.18-1.72)	<0.001	0.272	
**Hemodynamic goals**								
**HHA (≥5/6 goals)**	1.92 (1.38-2.68)	<0.001	0.171	0.278	1.41 (1.13-1.75)	0.002	0.21	0.294
**SBP ≥90 (mmHg)**	1.81 (1.5-2.17)	<0.001	0.335	**0.007**	1.5 (1.22-1.85)	<0.001	0.226	**0.01**
**MAP ≥65 (mmHg)**	1.82 (1.51-2.19)	<0.001	0.547	**0.038**	1.47 (1.2-1.81)	<0.001	0.323	0.082
**PCWP<18 (mmHg)**	1.74 (1.33-2.28)	<0.001	0.105	0.618	1.32 (0.94-1.86)	0.115	0.088	0.743
**CI ≥2.2 (L/min/m^2)**	1.68 (1.39-2.02)	<0.001	0.649	0.926	1.44 (1.15-1.8)	0.001	0.865	0.953
**CPO≥0.6 (W)**	1.71 (1.42-2.05)	<0.001	0.487	0.822	1.41 (1.14-1.75)	0.002	0.707	0.733
**CPI ≥0.32 (W/m^2)**	1.78 (1.49-2.14)	<0.001	0.436	**0.037**	1.48 (1.2-1.83)	<0.001	0.603	**0.022**
**CPI_{RAP} ≥0.28 (W/m2)**	1.69 (1.4-2.04)	<0.001	0.763	0.665	1.42 (1.14-1.77)	0.002	0.717	0.605
**PAPI ≥1**	1.84 (1.5-2.27)	<0.001	0.967	0.42	1.46 (1.15-1.85)	0.002	0.741	0.718
**RAP <12 (mmHg)**	2.56 (1.71-3.84)	<0.001	0.566	**0.014**	2.03 (1.22-3.38)	0.007	0.239	**0.019**

P for interaction significant values: SBP <90 mmHg for non-achievers in this goal unadjusted HR 0.86 (0.53-1.42) P=0.56 in adjusted HR 0.59 (0.29-1.18) P=0.138. For MAP <65 mmHg in this goal unadjusted HR 1.06 (0.67-1.67) P=0.801. CPI <0.32 (W/m^2) in this goal unadjusted HR 1.04 (0.67-1.64) P=0.851 in adjusted HR 1.02 (0.54-1.94) P=0.945.RAP <12 mmHg in this goal unadjusted HR 1.51 (1.25-1.83) P<0.001 and for adjusted HR 1.34 (1.07-1.64) P=0.008.

References

1. Kataria R, Kanwar M. Congestion in AMI-Cardiogenic Shock: Rethinking Treatment Goals. *J Card Fail* (2022) doi: 10.1016/J.CARDFAIL.2022.12.003

2. Mathew R, Fernando SM, Hu K, Parlow S, Di Santo P, Brodie D, Hibbert B. Optimal Perfusion Targets in Cardiogenic Shock. *JACC: Advances* (2022) 1: doi: 10.1016/J.JACADV.2022.100034

3. Baldetti L, Pagnesi M, Gallone G, Barone G, Fierro N, Calvo F, Gramegna M, Pazzanese V, Venuti A, Sacchi S, et al. Prognostic value of right atrial pressure-corrected cardiac power index in cardiogenic shock. *ESC Heart Fail* (2022) 9:3920–3930. doi: 10.1002/EHF2.14093
